# Analectic

**Published:** 1832

**Authors:** 


### Miscellaneous Intelligence.

### ANALECTIC, ANALYTICAL, AND ORIGINAL.

### ANALECTIC.

1. *Wound of the Head.—Ball extracted from the Brain.*—"D. M. aged 27, a soldier in the service of Bonaparte, was wounded on the 18th June, 1815. After lying three days on the field without tasting food of any description, he was taken to a village, and afterwards, to one of the churches of Brussels, without any thing having been done for his wound. On the 30th, I sent him, with many others, to the Gens d'armerie Hospital, and on the 4th July, he was placed under my immediate care.

"A musket ball had entered at the anterior portion of the squamous suture of the right temporal bone, and, passing backwards and downwards, fractured in its course the parietal bone, and lodged itself in the substance of the brain. There was a considerable degree of tumefaction of the soft parts, surrounding the wound, but, with the exception of a slight headache, and partial deafness of the right ear, he seemed to enjoy perfect health. He slept well, his appetite was good, his belly open, his tongue clean, his skin cool, and his pulse 72 of natural strength.

"On the morning of the 5th, the wound was laid freely open, when three large and several small pieces of bone were removed; and the ball which was found lodged in the posterior lobe of the right hemisphere of the brain, where it rests on the tentorium, was extracted without difficulty, and with small portions of the substance of the brain adhering to it.

After the wound had been carefully cleared of blood and small pieces of the brain, its lips were brought together and retained by two ligatures, along with adhesive straps, compress, and bandage. His whole head was kept constantly wet with cold water, a brisk purgative was administered, and he was placed on a very spare diet, with a small allowance of ripe fruit.

"Under this management, (a laxative being daily administered) he continued free from pain, or any derangement of the system, until the 16th, when he complained of lancinating pains through the back part of his head, of uneasiness from the light of a candle, and from noise. The wound looked remarkably healthy, with only a small discharge of healthy pus, and all that part which had been laid open by the knife was united. The pulsation of the brain could be readily discovered at two different points, where the large pieces of bone had been extracted. A brisk cathartic speedily removed these untoward symptoms, which there was reason to believe had been produced by some of his fellaw patients having indulged him with part of their allowance of food, the impropriety of which was distinctly explained, and means used to prevent the repetition of a similar irregularity. This, however, was no easy matter, as his appetite was keen, and he was confined to a very spare diet, viz. a small allowance of bread, with water whitened with milk, and sweetened with sugar.

"He now continued to enjoy his former good health, and nothing particular occurred till the 24th, when, on going my usual rounds, betwixt 10 and 12 o'clock, P. M. he called me, and said he was not well; the expression of his eyes was new and peculiar; and, along with the fulness of his countenance, evidently indicated a great and general irritation of the system. His pulse was, for the first time, 96, and hard; his skin hot and dry; along with a degree of stupor, and disposition to sleep. Upon making inquiry, I found that the medical officer (a young gentleman who had recently entered the service,) under whose care he had been placed for the last four days, had omitted to give him his usual laxative, and instead thereof, had that day allowed him *wine*, an *egg*, and other *extra articles*.

"A brisk cathartic was again immediately had recourse to, and after its operation a diaphoretic mixture, which, with a rigid adherence to his former mode of treatment, soon restored him to his previous state of convalescence.

"On the 5th of August, when I saw him for the last time, his wound was cicatrized. The pulsation of the brain was still visible, but with the exception of a slight degree of giddiness on stooping, he enjoyed perfect health. This he expressed by saying, that from his sensations he could not know that he had ever been wounded. It is proper to add, that, during the time of the above cure, he was allowed to smoke tobacco whenever he felt inclined, and which was almost constantly. It was never observed to produce any bad effect, and he argued the necessity of using it by saying, that it mitigated the otherwise almost irresistible urgency of his appetite, and thereby enabled him the more easily to comply with the very restricted regimen that was enjoined him, and which, as he was at length convinced, was essential to his recovery."—*Hennen's Military Surgery.*

2. *Wound of the Head. Severe Injury of the Head, with Loss of Speech and other Nervous Affections.*—Captain B——, a particular friend of mine, was wounded by a musket ball in the head at Waterloo, on the 18th of June, 1815. On the 19th, he was brought into the city of Brussels in charge of a medical officer, who gave me a most melancholy account of his case. On approaching the wagon in which he was conveyed, I was insensibly attracted to that part of it where he was stretched, by a low protracted moan, as of a person in extreme pain, but very weak. On calling him by name, he sat up, caught me by the hand, which he kissed most fervently, pointed to his head, and then to the site of a former wound which he had received at the storming of Badajoz, in 1812, from the effects of which I had the good fortune to relieve him. He then burst into tears, but without having the power of uttering a distinct word. His countenance was pale and ghastly, and his mouth somewhat distorted; his eye languid, and suffused with blood; his skin dry, but cool; his pulse about 90, soft and compressible. As I found that he had been bled on the field, I found myself contented with providing him a billet, and giving him in charge of his medical attendant, with directions to examine the wound most particularly; to enlarge it if fracture to any extent appeared; to administer a brisk purge, and to watch most carefully the approach of inflammation. The wounded being now poured in by hundreds, I was unable to see him before the 21st; his case, however, was reported to me daily. Much coagulated blood, and some particles of sand on which he had fallen, together with a thin scale of lead, obviously a bit of split musket ball, had been removed. His pulse had risen on the night of his arrival to about 100, hard and bounding, and he had been copiously bled in consequence. A cruciform enlargement of the wound had been made, which bled copiously, and gave a view of an extensive fracture of the left parietal bone. On my visit I found him nearly as follows:—Countenance pale, expressive of great pain, referrible more to mental than corporeal suffering; mouth still distorted; eye sunk, but its pupil dilatable; the power of articulating any distinct sound lost, but the desire obviously strong; pulse 80, soft: tongue clean, bowels open, (by saline purgatives;) urine copious, and with a rose-colored sediment; skin moderately warm, and at the region of the liver bathed in sweat; the liver itself obviously projecting, and giving a painful sensation when pressed upon, evinced by his wincing from the touch.

On examining the wound of the head, I found an extensive radiated fracture, occupying almost the whole of the left parietal bone; at the centre there was a piece of bone, apparently the size of a musket ball, beat in through the membranes of the brain, and bedded in its substance, but considerably more toward the frontal region than the occipital. The unequal pressure I found to proceed from a musket ball which was wedged in between the displaced pieces of bone and the portion, which, though cracked, preserved its situation. The separated piece was obviously much more extensive on its internal face than externally, and could not possibly be extracted without the operation of trephining, to which I proceeded. The leaden wedge, and several loose splinters which jammed it in, were easily removed; and on making one perforation with a large trephine, I removed the depressed portion of the bone, which was forced into the brain nearly an inch and a half from the surface of the scalp. It was of an irregularly oval shape, about one inch long by half an inch broad, and fractured in such a manner, that the internal table formed a much larger part of its circumference than the external.

No relief followed the operation; he passed an extremely restless night, and the pulse rose so rapidly and so high, that the abstraction of 16 ounces of blood became necessary. His breathing during this momentous night became, for the first time, perfectly stertorous; and, when I saw him in the morning, his whole appearance indicated the most extreme danger. He lay *coiled up* in the bottom of his bed; the right arm stretched out, and occasionally convulsed; no exertion could get a sight of his eyes, or his tongue; the mouth was more distorted than usual; the skin was nearly as on the day of the operation, except that the partial sweating over the hepatic region was increased in profuseness, and he seemed to wince more on pressure at that part; indeed, all the sympathies seemed to be entirely merged in those connecting the brain and liver. The stomach participated remarkably little, for he had scarcely any vomiting. His pulse alone gave me some hopes; it was nearly natural. On addressing him, he made an effort to rouse himself, but almost immediately relapsed into his former state. I directed a strict watch to be kept over him, and as my duties called me again to that part of the city where he was lodged, I visited him about midnight and found that a spontaneous billious diarrhœa had come on, and that he was much more sensible. He made an attempt to articulate, and pronounced audibly the letter T once or twice. The next morning, being the 5th from the receipt of his wound, his general appearance was amazingly altered for the better, the diarrhœa still remained, and his efforts to speak were continual. On the sixth day he grasped my hand with great fervor, looked piteously in my face, and to my inquiries as to his feelings, he uttered audibly, though with much labor, the monosyllable "THER," to which, in the course of the day, he added "O;" and for the three next days, whenever addressed, he slowly, distinctly, and in a most pathetic tone, repeated the words, "O; THER: O; THER:" as if to prove his powers of pronunciation. His general appearance, during all this time, amended considerably, and my hopes now began to revive. I therefore resolved to write to his family, and, before doing so, I printed in large characters on a sheet of paper, the following words, "SHALL I WRITE TO YOUR MOTHER?" that being the wish which it appeared to me he so long and ardently had labored to utter. It is impossible to describe the illumination of his countenance on reading these talismanic words, he grasped my hand with warmth, burst into tears, and gave every demonstration of having obtained the boon which he had endeavored to solicit.

From this period his mental faculties gradually developed themselves; he regained a consciousness of the circumstances immediately preceding his wound, and, in succession, of those of a more remote period. The power of speech was the last which he perfectly regained, and for which he usually substituted the communication of his thoughts and wishes in writing. Throughout the whole of his convalescent state, melancholy ideas constantly predominated, although, previous to the accident, he had been remarkable for his flow of spirits. He returned to England, nearly recovered, on the 29th of September, or 103d day from the wound.—*Hennen's Military Surgery.*

3. *Wound of the Thorax.—Penetrating Sabre Thrusts.*—"George Harman, aged 33, now hospital sergeant of the 10th hussars, received a wound through the lungs from the thrust of a sword, in an affair with some French cavalry near Morales in Spain, on the 2d of June, 1813. The sword entered the thorax behind, close to the basis of the right scapula, about the middle of its margin, and the point came out on the edge of the sternum, betwixt the articulations of the 3d and 4th ribs of the same side. He immediately fell from his horse, and soon fainted from loss of blood. In a short time he recovered, and had power to raise himself, and to sit upon the side of the road where he fell. In his removal to the village of Morales, about one hour and a half afterwards, he again fainted from returning hæmorrhage. When he had remained quiet a short time, the hæmorrhage nearly ceased.

"I examined the wounds, (says Assistant-surgeon Rogers, who has favored me with the case,) and found that situated near the scapula rather more than an inch in extent; that in front was scarcely half an inch. On inspiration, the blood was thrown out from the posterior wound to the distance of several inches, in drops, so as to sprinkle my face when examining it; it was also forced out of the anterior wound in a frothy state; blood was thrown up by coughing, the pulse was barely perceptible; a cold sweat had broken out: he was extremely faint, felt great anxiety, and complained of much pain in the chest; no appearance of emphysema at either wound. The edges of the wounds were united with adhesive plaster, and covered with a compress of lint, and a bandage applied. This was about three o'clock. At six in the evening the pulse had risen a little, the pain in the chest had increased, but no farther hæmorrhage had occurred. At nine o'clock there had been a return of hæmorrhage, not very great, and it had now stopped; the pulse continued much the same. At six in the morning of the 3d, I found he had passed a very restless night, but without any return of hæmorrhage: his pulse was quick and small; pain in the chest remained the same; respiration more difficult. At nine o'clock no change of symptoms; the bandage and dressings had become loosened; no emphysema round the larger wound; the edges of the smaller one were rather puffed; but the little tumefaction which appeared proceeded chiefly from blood extravasated. The wounds were again dressed, and I left him in charge of Mr. Pulsford, assistant-surgeon of the 18th hussars, to be removed to the general hospital at Toro. He now (January, 1817,) feels no inconvenience on moderate exercise; but running or any violent exertion causes quick and painful respiration. I have one other remark to make on his present state. If the finger be applied to the site of the posterior wound, a singular vibration is very perceptible when he speaks, confined immediately to the spot. If it be argued that the sword did not penetrate through the chest, but that the wounds were by separate thrusts, I can speak positively to the contrary. This being the first time we met the French cavalry, curiosity led me forward with the squadron which charged. I was close in the rear, and saw this man wounded, *after the enemy were broken,* being scarcely twenty yards from him at the time." So far Mr. Rogers; and I am myself enabled to add the following fact:—When I took charge of the hospital at Toro, on the 9th of the month, seven days after the action, I found Harman, who was an active acute man, giving every possible assistance to the other wounded, both English and French, and performing the duties of a hospital sergeant; no other dressing had been applied to the wound but a slip of adhesive plaster; and no morbid appearance whatever had taken place. He had suffered a convulsive fit of vomiting on two occasions after the wound was healed, without any apparent cause, in which he had ejected a large quantity of bile. He had another of these attacks some months after, when the diaphragm was severely affected with spasm; but his general health when I saw him a few months ago was excellent.—*Hennen's Military Surg.*

4. *Wound produced by the discharge of a pistol within the cavity of the mouth.*—M. de R——, one of the king's body guard, laboring under a severe gunshot wound of the face, was conveyed to the hospital of Gros Caillou, during the winter of 1815. This individual appeared to be dying when I paid him my first visit, twelve hours after the accident. A pistol barrel, loaded with two balls, applied to the palatine arch, and embraced within its circumference by the lips and jaw, was turned slightly forwards at the moment of the discharge, from which resulted a very violent explosion in the cavity of the mouth, and the escape of two balls externally through the arch of the palate and the nose. The anterior half of the former was destroyed, and the bony septa of the nasal fossæ, together with the part separating these from the cavity of the cranium, were broken into three pieces. The exterior surface of the nose was divided into three flaps, a middle one formed of its extremity, and the septum beneath, and the remaining two by the alæ of this eminence, and the corresponding portion of the lip. The middle of the latter was totally wanting, and a void space of about two inches in circumference existed; its edges were ragged and turned over. The velum palati and base of the tongue were cleft in a parallel direction from before backwards; and the left wall of the mouth presented an opening filled with coagulated blood. Every part of the face and neck was tumefied, and contained spots of ecchymosis, and vision was obstructed in both eyes, in consequence of the swollen condition of their lids. The remaining sensitive functions were also suspended. The pulse, almost entirely absent, was nervous, and the patient in a permanent state of convulsion and anxiety; in short, every thing induced us to fear a speedy dissolution.

It was, however, a matter of urgency to examine all parts of the wound, for the purpose of extracting foreign bodies, and simplifying it, as much as possible, with the view of causing the more or less exact reunion of its edges.

Having prepared suitable dressings, I proceeded, in the first place, to the removal of several bony fragments from the palate and nasal fossæ. Some portions of the edges of the wound were laid open, and those parts removed which were irregular, or had suffered from attrition. I afterwards inserted eleven points of the interrupted suture, in order to reunite with accuracy the three flaps of the nose and the uneven and very widely separated edges of the upper lip, taking care to include in the middle part the under septum of the nose. Several intervening sutures were employed, in order that the most exact relation might exist in every portion of the wound. With the view of promoting the approximation of the two maxillary bones, I fastened a platina wire around the two canine teeth, where the loss of substance ceased. Two pieces of large gum elastic catheters were introduced into the nose, to the extremities of which I attached thread for the purpose of maintaining them in this position. These canulæ, while aiding in the transmission of air for the purposes of respiration, contributed greatly to the preservation of the form of this organ. Small graduated compresses, placed on its sides and in the canine fossæ, and bound down and sustained by a retentive uniting bandage, completed this difficult and tedious operation.

Scarcely was it terminated when the patient experienced relief, and the nervous symptoms, indicative of tetanus, were immediately allayed. I took advantage of this moment of calm, to place him in a warm semi-bath, and administered, by means of a sucking-bottle, cooling anti-spasmodic drinks containing ice. Deglutition was performed with extreme difficulty, but by dint of care and patience, it was gradually restored. The night was passed in a pretty tranquil manner; on the following day, however, febrile action, which may be called traumatic, was developed, together with symptoms of head-ache and approaching suffocation. The application of a dozen leeches around the neck, and cups with scarifications to the back of the same part and to the thorax was with eagerness put in practice; the patient was also bled from the foot, and the same drinks and purgative enemata continued.

All the symptoms were speedily dissipated, and the individual recovered the power of his senses, with the exception of that of smell, of which he is destitute. The dressings were not removed until the eighth day; the wounds had united very uniformly and almost entirely. Some adhesive straps sufficed for perfecting their cicatrization; but an abundant secretion of pus, followed by the exfoliation or removal of several splinters of the proper bones of the nose, the ossa turbinata and a part of the maxillary bone, hollowed out by the balls, took place from the interior of the nasal fossæ and the arch of the palate. The destruction of the osseous vault of the nose produced an increased flow of tears in both eyes, with fistula lachrymalis, caused by the displacement or momentary obstruction of the nasal canal. The use of the catheters, of which we have spoken, the daily replacement of the bony fragments near the root of the nose by means of a sound, and injections into the puncta lachrymalia with Anel's syringe, re-established the course of the tears, and the fistulæ disappeared. The two maxillary bones also gradually approximated each other, so as to obliterate the communication between the mouth and nasal fossæ, and to render the possession of an *obturateur* by the patient unnecessary. In fine, after an attention of two months and a half continuance, M. de R—— was restored to his family in very good health, and without any sensible deformity.—*Baron Larrey's Memoirs.*

5. *Thoughts on Insanity.*—When the body is healthy and the mind sane, our beliefs, emotions, and actions are produced by mental processes, more or less complete in different individuals, but still in all by mental processes. We believe such a proposition because we have some evidence for it, good or bad; we experience angry or sorrowful emotions, because some thing irritating or depressing has occurred to our minds; we inflict punishment upon another from a vindictive emotion excited by a real injury; but in madness, these beliefs, emotions, and actions seem no longer to be the results of mental processes, but to be under the influence of a peculiar bodily state. I have conversed with those who have recovered from derangement, on the subject of their delusions, and have asked them what could have led them so firmly to believe such absurdities or impossibilities, what real or imaginary reasons they had; and they have told me that they had no reasons at all, that there was the thought in their mind, accompanied by the most undoubting confidence of its truth, but how it came there, they know as little as how it went away. Persons on the verge of melancholia will often declare that they are wretched, they know not why; that they have every thing to make them happy, and yet they feel no interest in life, a distaste for all their ordinary pursuits and pleasures, a wretchedness for which they can give no reason to themselves. In those extraordinary cases in which persons have committed murder on those who had never offended them, and towards whom they felt no antipathy, it seems that they were sometimes urged by some strange impulse totally different to the sense of injury, and thirst for revenge, which impels the sane man to commit such acts. If we are right in supposing that the instincts of animals consist of reasonable acts, not preceded by any reasoning process, but subservient to some bodily sensations in the animal, there would be a striking analogy between the two conditions, and insanity might be said to be the temporary conversion of human into animal nature. This has long appeared to me to be the most reasonable conjecture on this dark and mysterious subject.

It has been stated by legal authority, that to make out insanity, it is necessary to prove 'insane belief.' But what is the definition of 'insane belief?' Is it the belief of something either physically impossible, or utterly groundless and unreasonable? If so, it will apply indeed to the greater number of hallucinations, but will not reach them all.

I knew a captain of an East Indiaman who became deranged during a law suit about his father's will, and who believed that he had come into possession of £100,000 a year; he spent money lavishly, drove about the streets in a carriage, with a mistress, told me that he should restore the feudal system in all its glory, offered to give me a pair of carriage horses, and at length went abroad to dethrone the Grand Sultan, promising that if I went with him, he would make me his Grand Vizier, and give me a magnificent seraglio. In this case the predominant notion was utterly groundless and unreasonable; but what shall we say to those cases in which the predominant notion is such, that it is more reasonable to believe it than to disbelieve it, or is an actual truth? yet such cases there are.

In religious melancholy the patient thinks that he is the object of Divine anger. In this case is the prominent belief utterly groundless and unreasonable? Christianity, as represented to us in the New Testament, clearly leads to the belief that only a small part of the human race is to be saved: consequently the chances are much against any particular individual, and I know few persons who have not reason to doubt whether they have ever attained that strength of faith, purity of heart, and entire repentance, which are held out as the necessary conditions of salvation. When we consider the certainty of death, and the magnitude of the question, it seems more reasonable to feel anxiety and even terror about it than indifference; yet experience shows that people, as long as they keep their sound senses, bear the thought with sufficient lightness for all the uses of this world. Dr. Johnson, who seldom touched any subject without lighting on the truth, perceived this, as appears by the following opinion recorded by Boswell. 'Madness frequently discovers itself merely by unnecessary deviation from the usual modes of the world. My poor friend Smart shewed the disturbance of his mind by falling upon his knees, and saying his prayers in the street, or in any other unusual place. Now, although rationally speaking, it is greater madness not to pray at all, than to pray as Smart did, I am afraid there are many who do not pray, that their understanding is not called in question.'

Here the insanity consists not in groundlessness and unreasonableness of the predominant belief, but in its affecting the mind in a different way to what it does that of sane persons. I attended a deranged laly, whose predominant belief was, that her husband was unfaithful to her; the notion, so far from being unreasonable, was, I believe true, and she had known it for many years without any unnatural disquietude, but now it engrossed all her thoughts; she neglected her ordinary pursuits, took a dislike to her friends, felt no interest about her children, and sat silent and motionless from morning to night. After continuing deranged several months, she recovered, although she still retained the same opinion. In what, then, consisted her insanity? not in the groundlessness and unreasonableness of the predominant belief, but in its withdrawing her attention from all other thoughts and pursuits, in its overwhelming influence over her feelings and conduct.—*Gooch's Midwifery.*

6. *Treatment of Gout.* Read before the Royal College of Physicians, in June, 1831, by Sir Henry Halford, President.—So much has been written on the subject which I lay before you this evening, that I feel as if some apology were necessary for taking up your time with remarks upon the gout. But I rest assured that you will receive in good part the result of my long experience in the treatment of that disease; and that if I state to you that there is no malady to which I am called upon to administer, that I prescribe for with so much confidence in the resources of our art, as for gout, formerly the opprobrium medicorum, you will give me willingly a few moments of your attention.

I will not dwell upon the various seats of gout in the human frame. For though the terms Arthritis and Podagra would seem to limit the malady to the feet and the joints, we have seen it in almost every part of the human system. There are those who believe that they have observed it in the eye. I have certainly seen it in the kidney, in the urethra, and prostate gland, and in the tonsils. One of our esteemed colleagues has suffered it there; and I remember an eminent physician in the country so harassed by it, and so disappointed by finding no effect from the most approved remedies for the Angina Tonsillaris, that at length he plunged a lancet into it; if, peradventure, there might be some deep-seated suppuration there, to which he should give an exit. No matter followed; but in a few minutes the gout attacked the ball of his great toe. The Angina was soon forgotten, and the new disease ran its course with all its accustomed severity.

With regard to the remedies for gout, my dependence is placed upon the colchicum. Under the common circumstances of an attack of gout in the extremities, I do not use it immediately, but wait a day or two, until the malady shall have fixed itself. I then direct the wine of the root, prepared according to the directions of the Pharmacopœia; and I do not hesitate to declare, that I have not known a single instance of any untoward effect from it. It often cures the disease without any manifest increase of any of the excretions. Sometimes it produces perspiration, and sometimes it acts as a diuretic—the two objects aimed at generally by a physician in the use of our common resources in the treatment of this disease, but so far is it from being prone to purge the body violently as the eau medicinale often did, that I find it necessary, in most cases, to combine a small portion of the sulphate of magnesia with the wine, in the draught in which I administer it. The formula which I have found most useful is a saline draught, with camphor mixture, a dram of syrup of white poppies, and from 35 to 45 minims, not more, of the wine of the root of colchicum at bed time; to be repeated in the morning with 25 drops only of the wine, and half a drachm of the syrup of poppies, and in this dose a drachm of the sulphate of magnesia. It is necessary to repeat these draughts for three or four successive nights and mornings, and to follow its use by a pill containing three grains of an acetous extract of the colchicum (made by evaporating an infusion of the root in vinegar), and one or two grains of the Pulv. Ipecac. Comp., and the same quantity of the

Extractum Colocynthidis Comp., and to terminate the whole by a mild purgative.

It has been objected to the colchicum, that it produces a temporary good effect only, and that the gout is apt to recur when treated with this medicine after a shorter interval than usual. Be it so for argument sake—yet surely the weight of three or four attacks of the disease, of three or four day's continuance each, not more, is hardly to be compared with the pressure of a six weeks' painful confinement in the spring, and one of equal duration at the latter end of the year, as was the case before the value of this remedy was known; the paroxysms, moreover, terminating often by distortion and disfigurement of the joints by chalk stones—an evil which is now prevented almost universally by that control which the colchicum puts upon the inflammatory stage of a fit of gout. But my experience will not admit it to be true that the disease returns more quickly. On the contrary, when the liquid preparation has been followed by the acetous extract, I think I am fully justified in asserting, that the disease is removed for as long an interval as usually intervened between the fits when left, as it was left formerly, to patience and flannel.

I am not rash and inconsiderate enough to recommend this mode of treatment to you as a specific system for managing the gout in all its forms, and under all the circumstances of different constitutions, which may present themselves to you. The formula will require to be varied occasionally, and it may be proper in many instances of an enervated state of the frame to re-invigorate it by a light preparation of the Peruvian bark, after the colchicum has done its duty—or in other instances to give two or three doses of the Pil. Hydrargiri at bed-time every night, in order to recall the bile into its proper channels, if the colchicum or the sedative with which it has been combined shall have produced ash-colored evacuations by the bowels, denoting an obstruction of the bile.

Of all the preparations of this valuable medicine, I prefer the infusion of the root of Sherry wine. A preparation has been made, and is in frequent use, in the manner of an infusion of the seeds in preference to the root; but this has appeared to me to be apt to create an insupportable nausea, such an one as I have seen follow Wilson's tincture for the gout, and the eau medicinale. When such an effect has once followod, it is in vain that you request the patient to have recourse to it again. He will answer you, that he would rather endure his disease in all its severity than subject himself to the misery of such a remedy. This answer I have heard given to a proposal to administer the digitalis, when it had once affected the stomach in this manner—even when it had in one patient evacuated water from the chest in three successive attacks of hydrothorax; and in another controlled a dangerous affection of the heart for several years. No—these patients both declared that they would rather die than swallow one dose of digitalis more.

Before I dismiss the subject of colchicum, I must add that the use of this vegetable in gout is by no means *new;* for it is recommended by

Alexander of Trallas, a city of Lydia, in the sixth century, as a remedy for this disease, not under the name of colchicum, indeed, but of hermodactlys. Now the hermodactyls and the roots of colchicum are the same, as you will observe by a comparison of the specimens on the table. Being anxious to obtain some hermodactyls, I availed myself of the good offices of one of the king's messengers, and purchased those before you, in the market at Constantinople. They appear to be the same vegetable root as Sir G. Blane has stated on the authority of Sir Joseph Banks: though our estimable colleague, Dr. J. A. Wilson, is of opinion that there is a difference between them. I have not yet infused them in wine, but intend to do so immediately, and to try their efficacy upon gout in the same manner as I have prescribed the colchicum.

But is it not enough to state what I have found the most easy and effectual method of treating a fit of the gout, unless at the same time I lay before you the manner by which I attempt to prevent an attack.

As to medicine, I have had, incomparably, the most satisfaction in giving a few grains of rhubarb and double the quantity of carbonate of magnesia every day, either at bed-time or early in the morning; or, under evident weakness of the powers of digestion, half an ounce of the compound tincture of rhubarb with fifteen grains of the carbonate of potash, in some light bitter infusion daily, before the principal meal. The coarser purgatives should be carefully avoided; as I have often known a strong dose of physic, as well as a bleeding, aggravate a mere slight indication of gout into a severe decided fit.

But the management of himself and of his habits, on the part of the patient, is of more importance in keeping off this malady than medicine. His diet must be restricted, and he must dine at an earlier hour than is the custom at present among the higher ranks of society; his exercise must be gentle, but regular; his mind must be kept free from solicitude and care; he must avoid intense study, and he must be chaste. The word which Pliny uses to express this item of precaution is a remarkable one, and, as far as I remember at this moment, peculiar to himself—it is *sanctitas.* He remarks of a friend of his, a martyr to the gout, that 'Pedum dolorem fregit abstinentia, et sanctitate.' This point of conduct may be thought important in the eyes of the Roman, in consequence of what Hippocrates has remarked in the 30th Aphorism of the 6th Section, relative to the non-appearance of gout before puberty, especially as his own Celsus had adopted and recorded the same opinion. Ea raro vel castratos, ve pueros ante fœminæ coitum tentat.

Be this as it may, I venture to say that the caution is worth observing; for nothing enervates the system so much as this indulgence, especially in excess; and an enervated state of the body is that which renders it most assailable by gout.

I have only to add, that I have seen the best possible effect, in a great many instances, from the use of the waters of Aix la Chapelle, in restoring the healthy tone to the knees and ankles, enfeebled or stiffened by repeated fits of the gout.—*Bost. Med. and Surg. Jour.*

7. *Case of Iliac Aneurism with Ligature of External Iliac Artery.*—The subject of this case is a young man twenty-eight years of age, of good constitution; and the accident as far as the patient could judge was occasioned by a strain. The aneurism was situated above Poupart's ligament, which was raised by the lower part of the tumour, and by its indentation seemed to divide it in a large and small portion, the latter being below, the former above, occupying, apparently, a space equal to the lower half of the external iliac artery, and being as large and as prominent as a good sized orange; the pulsation very strong and distinct. During the month of the preceding operation, the patient was bled three times, the pulse, however, remained generally above ninety and sharp. There was a slight bellows sound at the heart, but the arterial system in other respects appeared sound. The situation and size of the tumor preventing the usual operation from being attempted, and it being probable that the greater part of the external iliac, if not the whole, might be unsound, Mr. Guthrie decided on performing the operation, so as, if necessary, to place the ligature on the common trunk of the right iliac arteries.

Mr. Guthrie commenced the operation "by making an incision in the side of the abdomen, extending from about an inch within the ninth rib, and about one inch above the level of the umbilicus, to nearly an inch within the ileum. This incision was six inches long, and the integuments, superficial fascia, and the external oblique tendon, were divided as rapidly as possible, and the fibres of the internal oblique were fairly exposed: these, being of uncertain thickness, were next divided cautiously, until the tendinous expansion of the transversalis was brought into view, going to from the sheath of the ureters. The lower portion of this was muscular, but I divided the tendon immediately above this part very easily, introduced a director upwards and downwards, and cut the transversalis upon it in both directions. The peritoneum was now exposed, covered, however, still by the fascia transversalis, which in places fastened and bound it down so firmly as to require to be divided with greater caution by the knife and director. This obstacle being removed, the peritoneum rose and fell with the intestines on each motion of the muscles of the belly, showing how carefully the preceding steps of the operation must be done to avoid doing it an injury, which at this part might be fatal. I now separated the peritoneum from the fascia transversalis and transversalis muscle, and endeavored to pass my hand behind towards the spine, between the ribs and the ileum; but I found there was not quite room for it to pass easily: I therefore enlarged the incision as much downwards as the proximity of the aneurism would permit, and divided the transversalis tendon upwards for about half an inch more. As it was here very firmly passing across, I raised the part to be divided on my two forefingers, and desired an assistant to divide it with a pair of blunt scissors: in doing this, a small fold of peritoneum was caught and divided, to the extent of near a quarter of an inch. It was, however, out of the way, and my hand could now pass with perfect ease, although it required to be done with the greatest caution, raising and separating the peritoneum before it, and passing over the psoas muscle, until my forefinger rested on the common iliac. It was only at this part of the operation that assistance could be given by any one: Mr. WHITE now, however, took charge of the bag of peritoneum containing the intestines, and raised it and them sufficiently to enable me to see the point of my finger resting on the vessel and on the bone beneath. I could now feel, and indeed see, the common iliac and its bifurcation into the internal and external iliacs, and it was as easy to have placed a ligature on any one as on the other of these three arteries, and more particularly on the common trunk. The external iliac appearing, however, to be sound, and of its natural size and appearance, an inch below the bifurcation, I cleared it with a blunt knife, and passed a ligature around it from within outwards, made of two strong threads of dentist's silk, well waxed. I then cut away one end, laid it straight, and allowed the peritoneum and intestines to fall back into their places. If I had a doubt about the soundness of the external iliac, I should have tied the common trunk; but as it was, and as I could with propriety place the ligature an inch below the bifurcation, I did it, and of course gave my patient an additional chance of escaping mortification.

"The wound was first brought together by three strong ligatures passed through the integuments, leaving the muscular wall to itself; and as these did not close the wound, four stitches through the skin, of a single thread each, were added in the interstices, which brought the edges of the whole line of incision into contact. The adhesive straps were then applied, and a compress was retained by other straps and a flannel roller. The legs were both raised by a pillow under the knees, the body bent, and the patient inclined towards the affected side.

The temperature of the limb previously to the operation, on Saturday, the 19th of November, was 102; the pulse ninety-two. On being put to bed, a hot bath was applied to the foot, and two persons were directed to rub the foot, leg, and thigh constantly under the bedclothes, which they did until midnight. At eight P. M., the pulse was 106, the temperature ninety-two. At 12 on Sunday, the pulsation of the femoral artery was distinguishable in the middle of the thigh, and he was free from pain in the limb, which I considered to be a most important sign of safety from mortification; for you may lay it down as a general rule, that, when a limb is going to mortify after such an operation, there is always great pain in the heel, calf, and even in the thigh, whilst at the same time there will be a mottled appearance of the skin, which, together, are the forerunners of an inevitable and generally fatal mortification. In the evening, the pulsation of the posterior tibial artery was just discoverable at the heel; and on the Monday, or forty-eight hours after the operation, I considered the danger of mortification to have passed by, and that of inflammation to have commenced. The pulse rose to 134, was jerking, but neither full nor hard. I had him bled to nine ounces, when the pulse diminished in volume nearly three-fourths, and he felt himself relieved from an oppression he could not describe before. The abdomen was a little swelled and tympanitic, but not painful any where on a fair degree of pressure. An enema, with ol. ricini, was given, and repeated with ol. terebinth, with full effect. In the evening the pulse was 140 and very peculiar, there being seventy strong beats, and seventy short feebler ones following them; the countenance a little anxious. I had him bled, with my finger on the pulse, until twenty-two ounces were drawn, when he became faint, a profuse perspiration came over him, and the limb lost its temperature.

"I may say to you now, in regard to the abstraction of blood, that it was very desirable not to produce positive syncope, yet to go sufficiently near it to repress inflammatory action; for, if syncope had taken place, it is possible the limb might have been lost. I was guided by this in the different bleedings which he underwent during the eight subsequent days, until 107 ounces were taken away in all; the blood, until the last, being cupped and buffed, and firm in the coagulum.

"He was kept on four ounces of bread and tea only each day, for the first fortnight, and on eight ounces, with a pint of milk, for the second. The loss of blood was not sufficient, even with the starvation, to subdue inflammatory action, and he took colchicum and digitalis also, until the intermission of the pulse, on the 3d of December, warned me to desist from their use.

"The ligature came away on the twenty-eigth day. The violence of inflammatory action induced me to suspect a greater degree of inflammation in the coats of the artery than was compatible with safety; and the removal of the ligature gave me great satisfaction, in assuring me of the soundness of the upper portion: the lower one seems to have inflamed, and the tumor, it would appear, partook of it also; for, on the 13th of December, it was evidently softer, and increased towards the line of incision, into which it discharged itself the next day, being thereby diminished in size. It still continues to discharge itself in a similar manner, but is so much diminished as to be scarcely perceptible, and I have every reason to hope will become consolidated with the surrounding parts.

"The man is now taking porter, fish, and beef tea; sleeps well; is free from uneasiness; and, as far as the operation goes, it has perfectly succeeded; and will form, I presume, a precedent likely to be followed in all similar cases.

"It is especially very worthy of remark, that the peritoneum seems scarcely to have inflamed beyond that part necessary for the cure, and that even this was confined to the outside. It was the general inflammatory disposition which required a decisive treatment, and not the local, which never exceeded its due bounds. I have said, when writing on the operation for placing a ligature on the aorta, that it must be done in a similar manner; and when my finger was resting on the trunk of the iliacs, I felt confident that I could with ease have placed it on the aorta. I laid bare one inch of the vessel: many persons saw it at a distance in the theatre, and, as the vessel does not exceed two inches and a half in length in the person, it is plain that very little more required to be done. I have no doubt but a further extension of the external incision for an inch upwards would have given quite sufficient space to have tied the aorta with ease; and I attribute the success of the operation on Hicks to the extent of the incision, which allowed of every step of the operation being done with facility."—*London Medical and Physical Journal, Jan.* 1832.

7. *Re-union of Divided Parts.*—Baron Larrey in his highly interesting volume of Surgical Memoirs, of which a translation has recently been published by Messrs. Carey and Lea, relates the following remarkable case of extensive division of the face by the cut of a sabre, and which was successfully treated by him. A Russian Colonel received from a French horseman "a stroke with a sabre, which cut off his nose at its base throughout its whole length. The instrument, being directed obliquely, had effected a division of the two canine regions, and two lateral parts of the upper lip, extending into the substance of the two maxillary bones on a level with the nasal fossæ. This division was bounded by the palatine arch, which formed a part of the flap, turned over upon the chin, and remaining adherent to the living parts of the face, merely by two small shreds of the upper lip, forming the commissures of the mouth. The entire extent of the nasal fossæ and the cavity of the mouth, without the alveolar arch, were seen on one side, and on the other, the flap formed by the whole of the nose, the upper lip, and the palatine vault, hanging over the chin. One of my pupils, finding this flap cold, and attached to the other portions of the face only at the two points, of which we have spoken, was proceeding to detach it entirely and dress the wound, according to the indications it presented, when I arrived at the bed side of the patient. I laid aside the scissors of the surgeon, and after examining the wound, arranged every thing for the purpose of employing the sature. I had some difficulty in removing the clots of blood which filled the nasal fossæ, and had been made hard by dust. I then detached the portion of the palatine vault, which adhered to the flap. It consisted of the anterior half of the superior alveolar arch. It had been separated from the remainder of the maxilla, on one side, between the canine and first molar, and on the other, between the first two molar teeth. I also detached from the flap several pieces of the proper bones of the nose, and ascending processes of the maxillary bones. The nose and lip were placed in their relative position, and I proceeded to re-unite them with the surrounding parts, by the interrupted suture, commencing at the root of the nose, and descending successively on its sides, the edges of which were approximated by ten parallel points of the sature. A piece of fine linen, dipped in salt water, was applied over the whole extent of the triangle, which was formed by the wound. I introduced into the nostrils two portions of large gum elastic sounds, for the purpose of preserving their form and diameter. They were commanded on the exterior by means of a a thread, which I inserted into their anterior extremities. Graduated compresses were placed on the side of the nose, and a retentive bandage terminated the dressings. I had the satisfaction to learn, on my return from Moscow, that this superior officer had perfectly recovered without any deformity. The cure of this case is remarkable, on account of the seriousness of the wound, and the few vessels which kept up a communication between the flap and the integuments of the face. Vitality was restored to the nose, and its re-union with the edges of the wound was exact and perfect."—*Larrey's Camp.*

8. *Irreducible Hernia.* By Wm. M. Fahnestock, M. D.—We had an opportunity, a few days since, of examining a hernia tumor of very great size, and of *fifty-five* years standing, which may be interesting as another evidence of the adaptive powers of various parts of the system to endure new burdens, which nature or accident occasionally throws upon a part destined for other purposes.

M. Brim, ætat. 79 years, a revolutionary soldier, and now residing near Waynesborough, Franklin county, Pennsylvania, first perceived a small tumor in the region of the internal abdominal ring, after severe exercise and straining labor, in the Highlands of New York State, 1777, which increased very much, and descended during the hardships and severity of the winter at the Valley Forge and the succeeding campaigns which achieved our glorious Independence. It continued to enlarge though still reducible for *thirty* years, during part of which time he wore a truss, but has not been able to reduce it for nearly *twenty-five* years, and now presents a very enormous and heavy tumor, measuring from the abdominal ring to the symphysis pubis, *two feet two inches.* The protrusion is principally on the right side, and appears to be chiefly intestinal; the penis is entirely buried in the tumor, and only discernible through a patulous orifice about the middle of the upper surface near the left side of the tumor.

A very singular circumstance attending this case is, that the subject has suffered but little pain from it; no severe pain, colic, or any symptom of strangulation having ever occurred; he has at all times eaten every variety of food with impunity; is generally very comfortable in his evacuations, seldom costive, occasionally flatulent, which distends the tumor very much and produces some uneasiness, but is immediately relieved on taking some stimulating carminative cordial, which discharges the wind with great force and violent commotion in the bag. The greatest inconvenience he experiences is, to sustain an equilibrium in walking, from the great weight of the tumor, which constantly tends to throw the body forward, and particularly on rising from a chair. By sitting on the ground or floor so as to relax the tension of the sac, he can manage to protrude the penis about three-fourths of an inch, and pass the urine with ease, which otherwise would act as a great irritant and excoriate the skin. He is in every respect a comfortable old man, with a flow of spirits and good humor, and delights in nothing more than fighting over again the battles of the Revolution, and recounting the hardships which emphatically tried men's souls.—*Amer. Journ. of Med. Sciences.*

9. *Symptoms of Aortic Aneurism.*—"When an aneurism is buried deep in the chest, and not capable of being detected by the sight or the touch, it does not present a single general sign which is peculiar to itself, and, therefore, pathognomonic of its existence. There are even cases in which it occasions no functional derangement—no inconvenience whatever; and the first circumstance that unveils the truth is, the sudden death of the patient, while apparently in the enjoyment of perfect health. We have met with six or seven instances in which large aneurisms had existed without awakening even a suspicion in the mind of the medical attendant. One, in particular, eluded the penetration of a distinguished foreign auscultator, though he explored the lungs with eminent success. We are acquainted with only one general sign of aneurism of the thoracic aorta which is unequivocal and certain, namely, a tumor presenting externally, and offering an expansive, as well as heaving pulsation, synchronous with the action of the heart. Of the remaining general signs a large class are identical with those of organic disease of the heart, viz. palpitation, dyspnœa, cough, tendency to syncope, terrific dreams, starting from sleep, hœmoptisis, livid or otherwise discolored complexion, cerebral or hepatic congestions, serous infiltration, &c. This identity arises from an identity of cause; namely, an obstacle to the circulation, which depends either upon the aneurism alone, or conjointly upon it and a disease of the heart, to which, sooner or later, the aneurism almost invariably gives birth. It is obvious, therefore, that the signs of this class are equivocal. There are, however, certain other general signs which are more characteristic: yet these even are of themselves ambiguous and unsatisfactory: as they only bespeak lesions of the viscera, or derangement of their functions, but do not proclaim the latent cause of the mischief. But when they coincide with the signs derived from auscultation, they lose their ambiguity, and rise into real importance; for the two classes of signs, general and stethoscopic, are a commentary on each other, and reciprocally borrow a precision and certainty of which they are individually destitute. We shall succinctly describe the general signs to which we refer, and subjoin to each the principal sources of fallacy. The means of detecting the latter we shall point out in the final summary.

1. When the tumor has attained a considerable magnitude, the cavity of the chest is preternaturally crowded, and the patient complains of a sense of constriction, infraction, and oppression, but these sensations are common to almost all diseases of the chest.

2. The radical pulses are sometimes dissimilar, or one is extinct; an effect dependent on obstruction or obliteration of the arterial innominata, or left subclavian. But the difference of the two pulses at the wrist, may proceed from a variety of causes independent of aneurism of the aorta, as, contraction of the origin of either subclavian from osseous, cartilaginous, stetomatous, or other deposition: obstructions in the course of the artery, occasioned by tumors, wounds, subclavian aneurism, &c.; an irregular subdivision of the humeral, brachial, or radial artery. We have known the most ludicrous surmises occasioned by the radial crossing to the outside at the middle of the fore-arm, and the superficialis volæ supplying its place at the wrist.

3. When the origin of either subclavian is contracted, the pulse at the corresponding wrist is a little later than the ventricular systole. We have not found this symptom uniformly present. The heart is more frequently its source than the aorta, and we have observed it to be most considerable in cases of regurgitation into the left auricle; but obstruction of the aortic valves may occasion it in a minor degree, particularly if this lesion is accompanied with extenuation or atony of the ventricular parietes. When the sign exists in both pulses, the presumption is strong that the source is in the heart.

4. According to Corvisart, a purring tremor—the *fremissement cataire* of Laenec—is sometimes perceptible to the hand at the middle or upper part of the sternum, and indicates aneurism of the ascending aorta. Purring tremor, above the clavicles, is an almost constant concomitant, and therefore a valuable sign, of dilatation of the arch; but, according to our experience, it is unfrequently and imperfectly occasioned by saccurated aneurisms, especially if lined by strata of lymph. We have never known the tremor to be occasioned below the clavicles by dilatation, unless the enlargement was so great as to extend beyond the lateral margins of the sternum, and allow the tremor to be felt through the intercostal spaces: but we have met with one case in which a dilatation of the pulmonary artery, though not voluminous, afforded a marked tremor between the cartilages of the second and third ribs of the left side: these, however, are not remarkable, as the artery *naturally* lies nearly opposite to the part described. We have never known a sacculated aneurism create a tremor below the clavicles, unless the tumor had eroded the bones of the chest, and presented externally underneath the integuments.

But the purring tremor may be occasioned in any part of the chest by mucous rattles, particularly those of the snoring kind, in the large bronchial tubes; and we have observed that, when derived from this source, it is a very common cause of deception, in reference both to aneurisms of the aorta and ossifications of the heart. Purring tremor of the pulse is regarded as a sign, though it is a fallacious one, of ossification of the aortic valves. From many dissections, it has appeared to us to be generally connected with two circumstances, viz. a powerful action of the heart, and ruggedness, without appreciable obstruction of the aortic orifice, or interior of the vessel. It, therefore, seldom exists, unless either the action of the heart be accelerated, or the left ventricle be hypertrophous.

5. When the trachea, or primary bronchial divisions, are compressed by an aneurismal tumor, a harsh wheezing, or sibilous sound, proceeding deep from the throat, characterizes the respiration, the voice is either croaking or reduced to a whisper, or it is a compound of both; the breathing is often extremely laborious; and when the heart is simultaneously diseased, dyspnœa sometimes occurs in paroxysms of the most suffocating severity. When the œsophagus is compressed, deglutition of solids is rendered difficult, and sometimes impracticable; for the descent of the morsel excites an excruciating pain from the summit of the sternum to the spine, or lancinating deeply in every direction through the chest.

But compression of the trachea or œsophagus, with the above symptoms, may be occasioned by tumors of any description. Wheezing respiration may proceed from an accumulation of glutinous mucus in the great branches. We have likewise known it produced in an extreme degree by laryngitis with thickening of the soft parts covering the arytœnoid cartilages, and also by ossification and ulceration of the larynx from strumous, syphilitic, and mercurial disease. So difficult is it to distinguish the seat of wheezing respiration, that it has in many instances, been imputed to an affection of the larynx, when it was in reality occasioned by an aneurism of the aorta; and bronchotomy has several times been actually performed with the view of obviating suffocation.

6. When the vertebræ are eroded, the patient suffers an intense terebrating pain in the spine; and when the brachial plexus of the nerves is compressed by the tumor, an aching sensation pervades the left shoulder, neck, scapula, and arm, with numbness, formication, and impaired motive power of the limb. But I have met with cases in which nearly similar pains were experienced, although there was no destruction of the vertebræ; and it is common to hear individuals affected with rheumatism or spinal disease make the same complaints. The affection of the arm may be occasioned by various forms of organic disease of the heart, and it thus constitutes a part of that concatenation of symptoms which are denominated angina pectoris. We have likewise often met with it in hysterical females subject to palpitation, and occasionally in cases of pericarditis. In all these cases, the pain probably originates in the irritation of the cardiac plexus of the sympathetic, propagated to the brachial plexus.

7. When, in consequence of an adhesion between the aneurismal sac and the pleura, the blood plays upon the lungs, a sense of ebullition is said to be experienced. But the same symptom is familiar to individuals laboring under phthisis, or chronic mucous catarrh: and it proceeds from the successive bursting of large bubbles, formed by the transmission of air through the fluid in tuberculous caverns, or in greater bronchial ramifications.

8. It occasionally happens that the patient suffers excruciating pain from a spasm, pursuing the course of the diaphragm, and binding the chest around, as with a cord. This symptom is too vague to be important, and it also occurs in hysteria, gastrodynia, colic, spinal diseases, and rheumatism of the diaphragm.

9. A pulsation is felt underneath the sternum, or ribs, at the superior part of the chest. This, although one of the least equivocal signs of aneurism, is not without ambiguity. It may be occasioned by a tumor of any description, as an enlarged gland or a cancer, interposed between the sternum and the aorta, and receiving the pulsation of the latter. Even Dr. Baillie says, 'But we are not to conclude from this symptom (viz. pulsation at the superior part of the chest)

that there is certainly an aneurism. I have felt the same kind of pulsation in other cases; as, for instance, where the pericardium was found strongly to adhere to the heart; where there was a slight inflammation upon the surface of the heart, with a little more water than usual in the pericardium; and where a morbid enlargement had taken place in the heart, without any aneurismal swelling.' Every one much conversant with disease must have made the same observations.

10. A pulsation is felt above the sternum or clavicles. But this may be occasioned, 1st, by enlarged glands, or other tumors seated on the subclavian artery, and receiving its pulsation; 2d, by varix of the jugular vein at its junction with the subclavian; both of which conditions have deceived expert practitioners; 3d, by subclavian aneurism. This affection sometimes resembles aneurism of the aorta so exactly, that it is extremely difficult to distinguish them. Allan Burns records a case in which all the eminent surgeons of the district were unanimous in pronouncing the affectian subclavian aneurism, yet it proved to be aortic. Sir Astley Cooper has published a number of similar cases, and one is mentioned by Professor Monro tertius. 4th, a pulsation above the sternum or clavicles may be occasioned by carotid aneurism. This, also, may readily be confounded with aneurism of the aorta, or of the subclavian artery. In April, 1826, we saw a case at Guy's Hospital, which led to much deliberation respecting the propriety of taking up the carotid above a pulsating tumor, supposed to be an aneurism of that artery. It was finally decided that the tumor was too low, and the design was judiciously abandoned. The affection proved to be a dilatation of the aorta and arteria innominata. The carotid was sound. This state of parts was indicated to us by the stethoscope. Mr. Hodgson met with a similar case.

11. The superior and middle parts of the chest are dull on percussion. But this sign is common to an infinity of other diseases, and the resonance is seldom impaired unless the aneurism be very large."—*Cyclopædia of Practical Medicine.*

10. *Partial Fracture of the Long Bones in Children.* By J. Hart, M. R. I. A., &c. &c.—At length the silent city, for the silence of its university is typical of itself, has put forth a medical periodical. Yet the effort appears to have been a great one; and Medicine alone has not yet felt able to shake off the lethargic drowsiness which oppressed her, without the friendly aid of Chemistry. The journal which has just seen the light in Dublin, and of which the first number is now before us, is entitled, the Dublin Journal of Medical and Chemical Science. It consists of 116 pages, 56 of which are devoted to original communications—17 to bibliographical notices, and the remainder to articles of miscellaneous intelligence. Whether it is to be monthly, quarterly, semennial, we are not officially informed, nor strange to say, do we find the usual prospectus, which tells how much a publication is wanting, and how lamentably deficient all existing sources of periodical information are acknowledged to be. We hail with pleasure our contemporary from the green isle, though, by the bye, *its* wrapper is of deep yellow, and need scarcely say that we wish it all success. It is at present too late to give an adequate notice of many of the useful papers it contains, and we must content ourselves with the first, because it is the shortest. It is on the partial fracture of the long bones, which is frequently met with in practice.

"The bones differ very much in the relative proportions of the animal to the earthy part entering into their composition at different periods of life, a circumstance which materially affects both their liability to fracture, and the length of time necessary for the accomplishment of re-union in individuals of different ages.

Thus, in infancy and childhood, the animal part of the bones bears a greater proportion to the earthy, whence they possess a greater degree of flexibility. It is owing to this that fractures rarely happen in young children, notwithstanding the many falls to which they are subject, before they have acquired the power of maintaining their equilibrium in their earlier essays in walking.

On the other hand, the bones of persons advanced in life, are harder and more brittle, in consequence of the accumulation of an increased proportion of the earthy part, to which is to be attributed the more frequent occurrence of fractures of the long bones in elderly persons especially.

As the bones of children are less liable to fracture from the cause assigned above, so is the process of their re-union more speedily accomplished, inasmuch as their growth being still in progress, the blood-vessels engaged in the function of their nutrition are in a more vigorous state of action; while in the case of older persons, whose bones contain a less proportion of the animal part, and more earthy or more saline constituents, re-union proceeds more slowly, because ossification being completed, the nutritious blood-vessels which were actively engaged in that process have fulfilled the task which was allotted to them, after which these vessels undergo a diminution in size, and a corresponding relaxation in their activity.

While the long bones of children are soft and flexible, they are still subject to a kind of injury, the occurrence of which is incompatible with the brittleness of the same organs in adults. This injury is a fracture which extends through a part of the diameter of the bone, the remaining part becoming bent in the manner in which a branch of a tree yields to an attempt to break it while it still retains its sap.

It has fallen to my lot to meet with five cases of this injury within the last three years, one of which occurred in the humerus, two in the radius, and two in the femur, and as this kind of fracture is not particularly described in any of our systematic works on Surgery, nor in any periodical publication to which I could obtain access, I shall briefly notice the particulars of one case, and conclude the paper with one or two remarks on it.

The diagnostic symptoms of this affection are very simple, they are the following: pain and a bent state of the bone injured, without absolute shortening of the limb; on the contrary, it is lengthened on the side to which the ends of the fractured part of the bone project. By attending to these circumstances it will be always easy to distinguish this injury from ordinary complete fracture.

*Treatment.* The first indication in the treatment is to straighten the bent bone; to effect this, much care and delicacy of manipulation are required, for if it be rudely attempted with a force too great or too suddenly applied, the part of bone which was merely bent may be broken, and the fracture rendered a completete one, the difficulty of treating which without deformity, will obviously be greater in a child, than in a person who can understand the necessity of submitting to the restraint.

The next indication to prevent the occurrence of the deformity, and to keep the fractured surfaces in contact until they become united by callus, and this is to be fulfilled by the judicious use of splints and bandages, as in ordinary fractures.

I need hardly remark on the shortness of the time (eleven days) in which re-union was completed in the above case: it illustrates the principles laid down in the commencement of this Paper, which refers to the rapidity of the re-union of fractures in children, owing to the more active state of the vessels engaged in the business of ossification at that period of life."

We have seen many instances of such complete fracture, and, unless our memory greatly belies us, have related some in a former Number of this Journal, though we have not leisure to search for them at present. However, we have twice seen this accident in the clavicle, once in the tibia, twice in the femur. We would particularly direct attention to a modification of fracture, probably of this description, which occurs in the bones of the fore-arm. We have seen three instances of what we are about to mention. A boy receives a fall, and in endeavoring to save himself, the weight of the body is received upon the outstretched hand. He applies to the surgeon, with the bones of the fore-arm bent either towards the palmar or dorsal side. They are obviously curved, nay, they even form an angular projection, and moderate force is quite ineffectual in restoring them to their proper shape. In each case we put the fore-arm across our knee, used considerable force of the leverage kind, heard the bones give an audible snap, and immediately were able to restore the limb to its natural shape. Under the application of proper splints and compresses, applied as for fracture of the fore-arm, for the usual time, we always succeeded in preventing all deformity and inconvenience. We mention these circumstance to put practitioners on their guard, as we have seen unpleasant mistakes occur.—*Med. Chirurg. Rev.*

11. *Rupture of the Kidney.* By Mr. Laidlaw, Middlesex Hospital—"October 11th, 1829. Alexander Elliott, a remarkably fine healthy young man, twenty-one years of age, while running at full speed, came unexpectedly in contact with the iron palisades of one of the squares, and struck his abdomen against them with such force that he was thrown to the ground with the shock: he immediately got up, and not feeling himself, at the moment, much hurt, walked home, a distance not greater than a hundred yards. Soon after he got home, he became very sick, and vomited, but did not consider it of much consequence, till about two hours afterwards, when, having occasion to make water, he found nothing but blood flowed from the bladder; and he then became alarmed, and procuring a coach, had himself conveyed to the hospital.

At the time of his admission, when I first saw him, he was pale, cold, and covered with a clammy perspiration; his countenance was anxious, and he was greatly agitated. Upon a superficial view, he appeared far more alarmed than injured. He complained of pain in the belly, particularly in the right hypochondriac region; his pulse was full and regular, at seventy-five. At his request some milk and water was given to him; but, immediately upon drinking it, he vomited it up. Twenty leeches were applied to the abdomen without delay. In the evening the symptoms became more alarming; the vomiting increased, and was accompanied with constant hiccup, which greatly distressed him, the pain in the belly was also much greater; he rolled about in the bed, was very irritable and restless; the anxiety of countenance was much increased, and the respirations were more frequent than natural: he did not complain when the abdomen was pressed with the hand. As he had not made water since his admission, I introduced a catheter, and drew off about ten ozs. of fluid, apparently chiefly blood, and directed that 15 more leeches should be immediately applied to the belly.

Upon visiting him early the following morning, (Oct. 12,) he said he was somewhat easier, but had passed a miserable, restless night, and had been greatly distressed by the constant vomiting; his countenance was still very anxious, and he appeared to be suffering a good deal from alarm and agitation. A catheter was introduced, and twelve ounces of bloody urine drawn off; this he said greatly relieved him. Towards night he became much worse; the belly was swollen and tense, and he complained of great pain upon the application of the hand; the vomiting was not abated, and it distressed him much more in consequence of the tenderness of the abdomen. It was found necessary to continue the use of the catheter, and the urine was deeply tinged with blood. Pulse small, sharp, and regular, its frequency not increased; tongue natural; skin hot and dry; and he complained of great thirst, which could be only partially allayed by moistening his mouth, as every thing he swallowed was instantly rejected. R. Hydrarg. Submur, gr. ij.; Pulv. Opii. gr. ss. statim. Haust Potassæ Citrat. quarta quaque hora.

For several days after the last report, every thing appeared to be going on favorably, and he seemed to be getting well rapidly, when suddenly, on the evening of the 21st October, he became much worse; his skin again became hot and dry; his tongue parched, and covered with a brown fur; and the anxiety of countenance, which was so apparent at first, had again appeared; he complained of great pain in the right lumbar region; the pulse was full and frequent, but regular. As his bowels had not been opened during the day, an enema was ordered, and he was directed to take the following powder every four hours: R. Pulv. Rhei, gr. v.; Hydrarg. c. Creta, gr. iij. M. fiat pulv.

On visiting him the following morning, (October 22,) I found him in less pain than the preceding evening, but the other unfavorable symptoms were unabated. In the course of the day he had a severe shivering fit, which lasted for several minutes, and the pulse became very small and quick. Upon examining the belly, the part originally injured seemed to incline to the side, and appeared to contain fluid. He was directed to continue the medicines and to have hot fomentations to the belly.

He continued in nearly the same condition during the following days (October 23d and 24th), appearing only to get a little weaker; but on the morning of the 26th, it was evident that there was no hope of saving him, as he was sinking rapidly. He complained of no pain in any part; he was perfectly sensible and collected, frequently saying that he had made up his mind to die; his countenance was sallow and very anxious, and he appeared to breathe with great difficulty; the pulse was too quick to be counted; the tongue was black and dry, and the skin hot, and without the slightest moisture. He fancied he should like some soda water, and it was accordingly given him, with small quantities of wine occasionally. At seven o'clock in the evening he died, having survived the accident just fourteen days. The body was examined eighteen hours after death. Upon opening the cavity of the abdomen, the intestines appeared of a dark sooty color, which was all that was remarkable in them. The whole of the solid viscera were in a healthy condition, with the exception of the right kidney, which, as had been anticipated, was found to be the seat of the mischief: by the injury it had been broken across just below the pelvis, and in it and the surrounding cellular membrane, a large abscess was formed, which contained about three pints of fluid of a light brown color: this was supposed to be pus mixed with urine."

This is a very interesting case in several points of view. It displays the series of symptoms which might reasonably be expected to follow a rupture of the kidney, and may afford a hint on the treatment most consistent with facts and principles. The general symptoms, in the first instance shewed that an important abdominal viscus was injured; the bloody urine told which that organ was. The earlier symptoms were restlessness, pain, not obviously and decisively increased by pressure on the abdomen, vomiting hiccup. What did they indicate? Commencing inflammation of the peritoneum? No, for the symptoms occasioned by the latter, which, to instance the most analogous case, we see after ruptured liver, intestine, or bladder, differ in many respects from the preceding. They indicated the injection of the cellular membrane around the kidney with blood and urine, and the inflammation thereby induced in it. The inflammation was in some measure checked, but on the 10th day other symptoms were developed; pain in the lumbar region, anxious countenance, typhoid fever, rigor, the almost unerring indications of the formation of matter. He died, and urinous abscess was found to exist in the cellular texture. Here, then, we see that in all injuries of parts surrounded by abundant cellular tissue, the great dangers to be apprehended are, the diffuse inflammation and the formation of matter, and that the latter may occur when the patient appears to be doing well, and the incautious practitioner is off his guard. We do not approve of the permission to the patient to drink milk and water immediately after the accident. If there is any one point on which we should be more particular than another, in cases of rupture or injury of the abdominal and pelvic viscera, it is the prohibition of both liquid and solid, food and physic, as far and as long as possible. We have had the treatment of many of these cases, and we were always most successful, in proportion as we strictly adhered to this maxim. We always endeavor to make the lancet and leeches supersede all other means for the first two or three days.—*Ib.*

12. *Cholera at Paris.*—The cholera is usually represented as having broken out unexpectedly and suddenly at Paris on the 26th of April last. This is not altogether correct. "Of those physicians," says the editor of the *Gazette Medicale*, "who unprejudicedly observed the development of the reigning constitution, there is not one who did not foresee and almost determine the precise period of the appearance of the cholera. It is especially permitted for us to say so, since, in June last, we pointed out the prevailing constitution as one of the forerunners of the epidemic. The disease was regarded by us as a necessary and inevitable consequence of the elements which we indicated. We will go further, we have had in our possession for at least three months accounts of several unquestionable cases of cholera, occurring under the observation of men of education and of unimpeachable veracity; but the fear of alarming a population already exceedingly uneasy, induced us to preserve silence until the disease has become more completely developed. This development it has attained with great rapidity. Since Monday, March 26th, the period at which the first well attested cases are said to have occurred, the disease has been going on increasing by tenfold."

Between the 26th of March and the 20th of April, 10,476 persons died in Paris of cholera; and it is said that upwards of 30,000 persons were affected with the disease, not including those who suffered from slight symptoms, evidently depending upon the epidemic constitution; as diarrhœa, borborygmi, cramps of the stomach, pains in the legs, followed by debility, &c.

The disease first appeared in the most crowded and filthy part of Paris, though eventually few or no parts of the city appear to have escaped its ravages, and its earliest and most numerous victims were the wretched inhabitants of narrow, filthy alleys, worn down by misery, debauchery, and privations of every description.

The following summary and treatment of the symptoms of the disease, which we translate from the *Archives Generales* for May last, is said to be the result of the examination of 6,094 patients admitted into the various hospitals of Paris, between the 1st and 18th of April. Of the number just mentioned, 3,673 died; 1,595 were discharged cured, or perfectly convalescent, and 873 remained under treatment.

"Symptoms. *First Stage.*—The individual, if at the time in good health, is seized with diarrhœa; this occurs in some instances very suddenly, the discharges being copious; in other cases the diarrhœa creeps on slowly. There is little griping, and no tenesmus. Soon after the liquid discharges from the bowels take place, a sense of weakness is experienced in the lower extremities, which is sometimes scarcely apparent, while in others it is so great, that the patient cannot account for the feeling of exhaustion so inexplicable by the symptoms under which he labors. Syncope is threatened on every movement of the body. In some cases there is an intense pain of the forehead, the peculiar character of which, the head appearing as it were constricted, and which causes to the patient great inquietude. By degrees anorexia comes on; nevertheless, in many cases, the individual is yet capable of following his customary occupations—frequently, also, the above symptoms, in persons of an energetic or careless disposition, attract little attention, and this leading to a false security, causes serious injury to the patients. This first stage of the disease continues one or two days, and frequently longer, extending sometimes beyond a week, and causing the utmost debility. It is very generally present, and is easily treated.

*Second Stage.*—This is marked by cramps in the limbs and vomiting. The stomach discharges, first the food contained in it, bilious matters are then thrown up, afterwards those of a serous character, which become mixed with whitish flocculi, giving to the discharges the appearance of rice water, or of gruel. The discharges per anum present similar appearances—at first solid, they quickly become fluid, bilious, serous, and finally are composed entirely of a sero-mucous liquid of a whitish color. So similar are the stools in appearance to the discharges from the stomach, that it is impossible, at first sight to distinguish one from the other. They have always a peculiar smell, which is acid, but at the same time sickening, and readily recognized when once perceived. It has some analogy to that of the vapor of iodine, or of chlorine. The sweat of the patient seems to present the same odor, and which, in the absence of other symptoms, is sufficient of itself to present the diagnostic. The discharges from the stomach are more or less constant and abundant. To these succeed cramps, affecting successively the feet, hands, legs, and arms—they invade even the trunk, simulating pleurodynia, partial peritonitis, and more frequently lumbago. The more violent and general they become, the greater is the danger of the patient—the exceptions to this statement are extremely few. The pulse increases in frequency, being from one hundred and twenty to one hundred and thirty in the minute; the extremities become cold, and the arteries lose their normal tension—the blood which flows through them scarcely distending their parietes. The secretions are suppressed, or at least they are suspended; the respiration is laborious, sometimes more frequent, and at others slower; but there is constantly a sensation of suffocation, produced by the constriction of the base of the thorax. The patient is restless, agitated, frequently prognosticating his speedy dissolution. The intellectual functions are unimpaired. The features of the face become sharpened, their ordinary expression is entirely destroyed; the eyes are bright, and the tongue pasty.

"*Third Stage.*—This is the stage to which the term *blue* has been applied, from the circumstance of the face and extremities assuming a bluish venous and very peculiar tint. To the phenomena of the preceding period, succeed now an extreme exhaustion; the skin becomes of a violet hue, the pulse extremely weak, frequently even entirely ceasing in the radial arteries. The respiration is deep and interrupted; the breath is cold, and has the peculiar odour already alluded to. The voice, which had exhibited some degree of alteration during the preceding stage, becomes now extremely feeble, frequently inaudible. The intellectual faculties still remain, nevertheless, the patient exhibits a carelessness or an apathy almost complete. The force and frequency of the cramps diminish; the evacuations from the stomach and bowels are less frequently repeated, the skin is bathed with a clammy sweat, and completely cold; the tongue itself is cold; the eyes half opened, present a bluish color, and ecchymosis, as it were of the inferior part of the cornea and conjunctiva; the pupil becomes dilated, the nose contracted; the face assumes a cadaveric appearance, and the limbs become stiff like those of a corpse. The skin is dry, and no longer presents its usual elasticity, so that a fold made in the skin of the neck or chest, remains permanent. During this period the patient dies without convulsions, or any apparent pain, and more frequently without the knowledge of those who surround him, so insensible is the transition from life to death, and so strongly does the living patient resemble the corpse.

"*Fourth stage.*—Re-action sometimes occurs spontaneously, as we are informed by physicians who have seen it in patients left entirely without medical aid. To produce re-action should be the aim of the practitioner, for it indicates a tendency to a favorable termination. The pulse regains an increase of strength, and re-appears in the extremities; the coldness of the surface is diminished; the skin loses its violet hue; the conjunctiva becomes injected; the voice becomes more sonorons; the tongue and breath acquire their usual warmth; the respiration is more frequent and easy. A hiccough sometimes occurs, as though the diaphragm, in resuming its functions, experienced a difficulty in their proper execution. There is no longer either diarrhœa, vomiting, or cramp. Frequently, also, as the circulation becomes more free and vigorous, congestions of the brain take place; the head becomes red, and the patient may then sink rapidly. In other cases the only degree of re-action is of a more natural character, and the recovery of the patient is more speedily accomplished. Such is the general course of the cholera morbus as it presented itself in Paris. Many varieties, however, were observed in its phenomena, referable to the particular condition of the patient's system. Thus, in children, females, and very irritable persons, a form of cholera was observed in which the nervous symptoms predominated; the cramps were attended with true convulsions; symptoms were even observed which simulated tetanus; during the paroxysms of which the patient expired. In plethoric subjects, with large and robust bodies, the inflammatory form of the disease manifested itself more frequently; the tongue was red and irritated, the epigastrium was the seat of acute pain; there was violent fever, very copious vomiting; insatiable thirst, and other symptoms demanding evidently an antiphlogistic treatment. In other instances the asphyxial type predominated—the blueness of the skin occurred from nearly the commencement of the attack, and the death of the patient took place often very promptly.

"TREATMENT.—Notwithstanding all that has been said upon the existence in cholera of a gastro-enteritis or gastro-cephalitis, it is certain that in many cases, even of the most violent character, not the least morbid appearance is discoverable throughout the whole digestive tube. Sometimes we find in the small intestines a number of either isolated and tolerably prominent follicles, or of celulated patches, having the same appearance as those found at a certain stage of ordinary typhoid affections. In some cases, much more rarely, we find traces of gangrene in the mucous membrane, or black spots which exhale a decidedly gangrenous smell. MM. Renauldin, Martin Solon, Andral and Louis have met with these morbid appearances in several instances. They are nevertheless exceptions, and cannot serve as a basis, upon which to found a correct idea of the etymology of the disease. What seems more evident is the alteration of the blood. The proportion of free carbon being double, and that of the coloring quadruple, to what they are in a state of health. The aqueous, albuminous, and fibrinous portions of the blood are almost entirely wanting—in consequence of which it assumes the pitchy consistency so frequently mentioned. If this, which is evidently the essential morbid change, be made the basis of the etymology of the disease, and consequently of its treatment, it may be demanded what relation does there exist between the affection to be removed and the remedies employed to that end. In the first period, when the digestive tube appears to be the principal point of fluxion, it is all important to oppose this congestion. Of course, local bleeding and soothing injections are indicated. Some practitioners have employed other means; they have given ipecacuanha, and produced, in this manner, a sudden impression upon the whole intestinal tube. This shock has been salutary in a great number of cases; but it should not be concealed, that in other instances it appears to have had an injurious effect.

"In the second period, the internal congestion is more extensive; it already impedes the play of many of the functions, and the necessity of directing our efforts to its removal, increases rapidly. We may yet bleed if the pulse continues, and the patient is robust. Antispasmodics and hypnotic remedies, capable of arresting the progress of the nervous affection, which is now added to the first should likewise be administered. Iced drinks, even ice itself, are a very excellent means for arresting the vomiting. Injections, decidedly astringent, will succeed also in arresting the diarrhœa, and preventing that exhaustion which so rapidly follows discharges from the stomach and bowels. The administration of ipecacuanha even in this stage of the disease, has appeared to be attended with good effects, in bringing back the natural secretions to take the place of those produced by the morbid stimulus. Under the effects of an emetic of this substance, the bile has been known to be copiously discharged; the white matter of the dejections to be diminished, and shortly to disappear; the bladder to become filled with urine, and the cramps to cease.

"With respect to the third period, a new order of phenomena predominate. The coldness of the extremities, the feebleness of the respiration, the diminished action of the heart, all indicate a complete perversion of the functions of the organs. Even supposing, that during the first stages of the disease, some portions of the mucous membrane of the digestive tube should become the seat of a slight phlogosis, it does not follow, that this should demand the entire attention of the practitioner, now when life itself is on the point of ceasing, and the actions of all the principal organs arrested. Under such circumstances the subtraction of a small quantity of blood, is to say the least, useless, the impulsive power of the heart being almost destroyed, and the quantity of the circulating fluids greatly diminished. Our efforts should, therefore, be directed to the organs which are still capable of feeling the influence of the impressions we desire to make upon them —the stomach, in this case, is one of the points the most accessible to our remedies. The most diffusible stimulants, as ether, ammonia, &c. will now be found to produce salutary effects, and give to the patient some chance of recovery. Even at this period vomits have been administered, and the spinal marrow has always been strongly stimulated. These two means have had the effect of rousing the nervous influence on the point of disappearing—of calling back the powers of life, and of producing re-action. It will readily be perceived that a crowd of analogous measures are all equally calculated to produce these important results. We must say, however, that in this stage the chances of success are but trifling.

"Re-action once attained, we have to watch its progress and obviate any morbid effects it may occasion when not maintained within due limits. The convalescence from cholera is long and painful. It requires a constant watchfulness on the part of the practitioner, and the utmost docility on the part of the patient. It is unhappily too true, that a great number of patients recovered from an attack of the cholera, but too soon abandoned to themselves, have from the errors in regimen they have committed, been suddenly destroyed. The extreme debility which succeeds to the enormous discharges that take place in this singular disease, predisposes to serious organic affections—the most trifling pneumonia will in an instant become mortal—the slightest irritation of the stomach, will be at once accompanied with all the symptoms which characterize the typhoid diseases, and the patients sink the more quickly, as the plan of treatment proper in the latter affections cannot be put in practice in persons so completely exhausted."

13. *Remarks on the Pathology and Treatment of the Disease termed Malignant Cholera.* By J. P. Hopkinson, M. D.—"I saw no marks of inflammation except in cases of drunkards, or where it had probably existed antecedent to the attack of cholera; in one case inflammation was found in the caput coli; in another, the rectum was most violently inflamed, and sometimes the glands of Peyer were enlarged, but these appearances were by no means so general as to justify any conclusion as to their connexion with the immediate cause of death. Dissection had not as yet satisfactorily located the disease in the central nervous system. The lungs were almost universally healthy, and thus the abdomen alone remained to be studied in our pathological researches. As already stated no decided marks of inflammation presented themselves in any of the abdominal viscera—adequate to account for the sudden death, nor in fact are the symptoms during the course of the disease those of inflammation—but more of this presently. The appearance of the intestines externally is so peculiar, that no one who has once seen them, can be deceived. In every case I have examined, the most decided proofs of universal congestion were apparent. When the omentum was raised, the intestines, (generally congested,) presented a mottled surface formed of small bluish and red points, the former preponderating, so as to give altogether a dark chocolate appearance—a closer examination traced the connexion between these specks and the veins—of which the small ramifications, the branches and the trunks, were filled with dark blood. The mucous surface was more variable in its appearance. The valvuli conniventes being in some places of a bright red—in other places pale. *Ulcerations,* or *settled inflammation* I did not find in any case in the intestines. The inspection of the stomach was equally unsatisfactory, or perhaps more so. In some cases inflamed, in others not, sometimes having a soft mucous surface, easily scraped off, while in others again, this membrane retained its ordinary degree of firmness; in one word, the stomach, in this respect presented nothing I had not often seen, and what dissections made in various diseases do not reveal every day. The only condition at all general, was a congestion of the veins apparent on the external surface, and its being exceedingly large, flabby, and filled with fluids. The liver, kidneys, &c. although sometimes congested with dark blood, or perhaps disorganized by some old affection, showed no marks of recent organic disorder that were appreciable; bile and dark blood sometimes flowed when incisions were made into the liver, and there was invariably a total suppression of the secretory action of the kidneys; when the papillæ of these glands were compressed, a milky fluid exuded from the orifices of the tubuli uriniferi. The bladder was empty and contracted. The spleen had nothing remarkable in its appearance: there was no bile in the intestines.

Thus it would seem, since the morbid conditions of the nervous system are too abstruse for our detection, that nothing has been gained by the postmortem examinations, if it be not that a universal venous congestion pervaded the whole abdominal cavity, accompanied by a suppression of the natural secretions. It then appears probable, that the cause of cholera, whatever it may be, by diminishing the heart's action, and thus lessening the activity of the arterial circulation, and by producing a torpid venous circulation, perhaps the result in part of the former, perhaps produced by the direct observation of some morbid poison on the nerves, operates mainly upon the abdominal viscera; here it is, as we know, that the proximate causes act in bringing on an attack; and here it is we find developed the most prominent symptoms.

Now let it be understood, that although this pathological view became the foundation of the practice hereafter to be explained, it is not put forth with so much confidence as to limit further researches into the nature of this strange affection. One thing only is insisted upon, that, whatever the remote cause be, that at this particular time, in some circumscribed locations, and not in others, predisposes to the disease termed cholera; the most prominent symptoms are developed in the abdominal cavity, and to that point must we turn our attention, would we arrest the approach of death. The coldness of the extremities, therefore, the profuse sweats, the cramps of the muscles, the cold tongue, the immoderate thirst, &c. are not to be regarded, except as mere symptoms remotely connected with the morbid actions that are going on in the interior of the body.

A natural consequence of diminished arterial circulation is diminished temperature—but if the cause that produced this feeble action still continue to operate at the centre, how vain must all efforts to arouse action be, when applied to the extremities.

Again, as regards the thirst, since there is neither increased heat nor dryness of the mouth, upon what reasoning or theory do we allow the patient the free, unrestrained use of ice or cold drinks. Is it because he demands them? and is nature always so true in her requests, that we should listen to her. Our patient may be indulged, perhaps, to his detriment; I have known the water and melted ice, that have been allowed to a patient at intervals during the space of an hour, to be thrown up from his stomach *nearly as cold as they went down.* There cannot therefore, be much increased heat in the gastric organ, and we have a right to conclude, that the effect of cold and moisture is not demanded, since evident *coldness and moisture* actually exist in the mouth, and in all probability the stomach is in a similar condition. To those who are disposed to doubt the correctness of the theory here advanced, and who, bound in the chains of former doctrines, cannot give up the impression of an augmented temperature in the stomach, causing the thirst—to all such, let me here make one remark—what is the actual condition of the patient's skin? and what are his feelings connected with that condition? Cold and wet, is the reply to the first—and insufferably hot, answers the second. Have we not then before our eyes, and under our fingers, a cold, moist skin, giving the sensation of oppressive heat?—a phenomenon as yet not understood, and must we of necessity reject the same idea as applied to the stomach; merely because we cannot explain it? Look at the collapsed, dying individual, with a skin like marble, covered with the morning dew—what are his sufferings if attempts are made to confine him under covering? Does he not resist with all his strength, and cry out he is burning with heat? With this fact before me, I am content to say, there is a total destruction of all the natural sympathies in this disease, and referring the thirst, the cramps, and the feelings of heat, to morbid nervous actions, stop my speculations here, and follow that practice which experience proves most beneficial.

The profuse sweats can hardly be called either a secretion or an excretion. Our knowledge of the capillaries is as yet too unsettled to establish any theory upon this point; but when we see those parts of the body most remote from the centre of the circulation, and most deficient in arterial circulation, pouring out these sweats more abundantly as they are more distant, is not the conclusion warrantable that they are a kind of exudation, coming perhaps from the veins, (which are visibly more congested in the same ratio of distance from the heart,) and not the result of any arterial excitement? Let us now only transfer our attention to the interior of the body—let us admit a condition of the alimentary passages, similar to that remarked upon the skin, and have we not at once, an explanation of the immediate cause and source of the rice water discharges? Here then we unite the various considerations that have become the basis of our pathology and our treatment of cholera.

The cramps cannot be easily explained. I feel a hesitation in offering a theory upon the subject, and shall defer any further remarks respecting them for the present.

Allowing the cholera to be a disease of a typhoid character—grant it to be connected with an almost universal congestion of the venous system, and not inflammatory, what are the indications? These appear to me to be several and distinct, and that the whole success of our practice will depend upon the manner and the order in which they are met. Let us first review the symptoms of a man in the state of collapse. He is either pulseless or nearly so. The extremities are icy cold, and checkered with large spots of limpid water, collecting as fast as they are removed; the fingers corrugated, the nails blue; the tongue and breath chilled, and the thirst painfully urgent. To these add in most cases violent cramps of the voluntary muscles, purging of an almost colorless fluid, and the rejecting from the stomach of every thing swallowed. Apparently the cold extremities, the vomiting and the cramps, are the most urgent, and therefore the first to be removed, and hence hot applications, frictions of various kinds, anti-emetics and opium, seem so natural a prescription, that it is difficult to resist their employment. The real disease is not studied; the symptoms alone lead every one astray. To relieve the spasms and check the vomiting and purging, nothing is more natural than large doses of opium. To restore the temperature of the skin, cold and damp, who would not resort to heat and stimulating frictions? The want of success in this treatment leads to the *trial* of something else, combining perhaps some one of those remedies, (generally opium,) or else excluding them all, and standing forth with all the presumption of a specific.

Let us now take up the indications under a different view of the disease. Supposing the venous congestion be proved to be the immediate cause of those symptoms, viz. that the morbid cause having diminished the power of the heart, (whether through the nervous system or not, we need not stop to inquire,) the arteries as a consequence carry less blood than usual, must not the remote parts of the body, under such circumstances, first feel the want of their accustomed supply? The coldness of the skin then is merely a symptom, an inevitable consequence, and as such it should not be regarded in our treatment. Again, grant that a diminished arterial circulation throughout the abdomen, produces there a state of things similar to what is witnessed without, ought not the same results to ensue, namely a diminished temperatnre, a loss of tone, and suppression of natural or healthy actions? How futile then all attempts to arrest the vomiting and purging, by ordinary means, such as opium, &c. These symptoms are merely the natural results of the grand cause that is still triumphantly operating, and if they, as such, are permitted to attract our attention, are we not thus decoyed away from the main object? If this position be true, the rice water discharges pouring from the rectum, and the fluids thrown from their stomach, are worthy of no more particular attention than the dew-like drops that collect upon the skin. But let it be remembered that the stomach is an organ, possessing a higher vitality than any other viscus of the abdomen, and that the detention and consequent loss of tone in it, must exercise a greater influence over the whole system, and on which account its immediate restoration becomes a matter of the first importance. I conceive then this organ to be in a state of *flabby distention*, if I may so speak, to have all its veins highly distended with blood going through a torpid circulation, its arteries contracted and enfeebled in the same ratio, and having a diminished temperature. If it be true, that through the medium of the stomach, we must hope to act upon the rest of the system, it behooves us first to prepare this organ for so important a duty; we must arouse its energies ere yet it be too late; we must bring on the tonic contractions of its muscular fibres; we must expel this black, venous blood, and endeavor to restore the arterial circulation to its natural superiority. What can do this but an *emetic?* We have no common cholera morbus to deal with, and would have nothing to fear in the use of such a remedy if we had. The choice of an emetic is, however, a matter of some consequence. The mustard I have never tried; all our common emetic medicines are either slow and uncertain, or else violent and dangerous. The Russian practice suggestsd the salt, and experience has proved it precisely what in every respect I would have it. My first course then is to dissolve two large spoonfuls of common salt in a pint of water as warm as the patient can bear it, of which a tumblerful is given at once. Almost instantaneous emesis results, and generally some retching follows; if however, these are not sufficiently well effected, that is, if it appear probable that the stomach has not completely contracted, another tumblerful is given and our object attained.

The evacuation of the stomach is not the only advantage derived from an emetic. Nothing in these cases excites the heart to action so certainly as the retching that accompanies the act of vomiting; where stimuli have failed to make the slightest impression, the effort of vomiting has instantly restored an extinct pulse. But most of all, this operation is followed by a less irritable stomach, and if caution be observed suddenly in regard to drinks, no vomiting of any consequence will return. Thus we accomplish the grand objects of the first remedy; and so far as I have yet observed, if the salt fail to excite vomiting, the case is desperate. Immediately after the emetic, perhaps in five, ten, or fifteen minutes, twenty grains of calomel for an adult, mixed with a little white sugar, are placed in the mouth dry, and washed down with some cold water. This medicine is given at this time, not with the expectation of any immediate effects, but with a view to its subsequent operation. Ten grains are then given at intervals of an hour, until often a dram or more is taken. If reaction come on, the calomel is immediately stopped, as every object will be attained when the system is so far restored as to receive its influence. It is then the ulterior effect upon the secretions that makes its early use important, especially as it does not interfere with the other remedies next to be mentioned. It is now clear that thus far only one indication has been answered, viz. that of restoring the stomach to a more natural condition. The next important object is, to do for the rest of the system what we have done for the stomach, and is, to overcome or remove the universal venous congestion, but of course by a different means. For this purpose there is no substitute for venesection, and the blood must be drawn *from the veins.* It will not do to open an artery; this exhausts the patient, but does not relieve the *venous congestion.* In this respect, perhaps, we have an exception to all other cases; *we must then open the veins.* If the pulse is not perceptible, or if it be very feeble, it is better to begin by applying cups over the whole abdomen, and then if the pulse rise, we may open a vein in the arm, or in the foot, and watching the pulse, let the blood flow until re-action, or the improved condition of the patient indicates the attainment of our object. As regards bleeding from the arm, unless the pulse be full and hard, and there are violent spasms, we should be cautious not to resort to this practice too indiscriminately. I have seen it do no harm where a vein has been opened before the habits of the patient or the real object of the operation has been consulted. Although the pulse from the beginning has in some cases been so good as to bear a free bleeding, it has generally been necessary to resort to cupping before the restlessness and anxiety, (attendant upon the abdominal congestion,) have been relieved. For, the tossing about, the feeling of fulness and uneasiness, seem to me all to depend upon this torpid congestion of the abdominal viscera. Although not always attainable, especially in country practice, a valuable adjuvant is obtained in the application of leeches to the epigastrium, but more particularly to the anus, for here we come directly to the point and draw blood from the abdomen. The next thing to be done, to retain the ground we have thus gained, is to apply a large blister over the epigastrium, and if we succeed in producing inflammation on the skin, (vesication is not necessary,) our patient may be considered in most cases convalescent. These are the grand principles of our practice founded upon the pathological view of this disease now offered to the medical profession in these remarks. But there are some other matters to be attended to, of no small importance.

1st. The nausea and occasional return of the vomiting; to relieve this, the effervescent draught, taken in a state of effervescence, and made in preference to fresh lemon juice or a little cold soda water, to which have been added ten or fifteen grains of sup. carb. soda, with ginger syrup, will prove most effectual. But do not let the patient indulge his desire for drink too freely. At this time it would appear that the pyloric orifice is obstinately closed, and absorption goes on so slowly in the mucous membrane of the stomach, that ultimate vomiting ensues as the only means nature has to relieve herself of an oppressive load.

2d. The thirst is so urgent, that it seems cruel to refuse the patient every drink, and yet it becomes our painful duty to restrain him in the gratification of his desire. With this view, ice is generally allowed, but in very small quantities. I prefer much limiting the patient to the use of cold water as a gargle, and have generally been highly gratified to see him, when made aware of the danger of swallowing so much water, amuse himself with both pleasure and relief in washing his mouth. The sensation of thirst, in this disease appears, as already stated, to be entirely a morbid nervous feeling. The tongue and mouth are cold and moist, a condition directly opposite to that generally accompanying thirst, and we have no reason to suppose that there is any increased temperature in the stomach to cause it, as the observation already made would go to prove, since as then remarked, the water which was thrown from the stomach was still cold. Would not hot drinks taken into the mouth, by restoring a natural action, tend to remove an unnatural condition?

If the patient be not an habitual drunkard, or have not any chronic affection of his viscera, this course will generally succeed in bringing on re-action, or to speak more correctly, in allowing re-action to come on. We first discover the doughy condition of the skin, replaced by a natural elasticity, so that if we pinch it up, it immediately retracts. This change is first noticed on the thighs and shoulders, whence it gradually progresses, to the remotest points, until life once more seems universally disseminated; at the same time the profuse sweats cease, and the surface, without becoming parched, as in a hot fever, presents a natural degree of warmth and dryness. The thirst also disappears, an effect, in part, perhaps, of the calomel, as I have seen it relieved before the tongue had regained its warmth.

3d. We said nothing respecting the constant purging in our treatment, because we consider it merely as a symptom. If, however, the stools are copious and frequent, more as a matter of convenience than from any idea of their suppression benefitting the patient, the following injection is thrown up the rectum:—To one pint of cold water, add, acetat. plumbi, ozj.—one-half to be first used, and if rejected, the remainder. This will arrest the purging. It is more than probable that it extends its influence further than the large intestines, with which it comes in contact, for it has generally been remarked, that no discharges have come away after the use of this injection, until the calomel makes its appearance, escorting the bilious matters, the harbinger of our patient's recovery. The stools, it may be well to remark here, which make their appearance from twelve to twenty-four hours, or even later, after the exhibition of calomel, are very peculiar. They are thick and of a bright green color; so much so, that they are familiarly termed the "spinage stools." The improvement of the patient after this takes place, is remarkable.

4th. Respecting the cold extremities, as already stated, we do not regard them as a matter of consequence, in the treatment of the *disease.* Warmth is generally applied to the feet, legs, and thighs, by means of bags of hot sand or salt, but with another view; nor have I, as yet had any reason to suppose they have ever assisted in bringing on re-action; yet it must be acknowledged that they aid in its accomplishment when once it has commenced, and if used in time may prevent or protract the approach of the collapse; for then there is still vitality enough left to feel their influence; but what can we expect from heat applied to a surface, as it were, dead and insensible? In fact, have we not all seen hot applications impart their heat, only as they would to a block of wood, which feels it not, and loses it as easily as it was received? An exception has been hinted at—it is in cases where the cramps are very severe—heat applied to the muscles, and dry rubbing with the hand have proved highly beneficial in relieving the cramps, which are often so distressing as to cause the patient to cry out. The relief is so marked, that he will tell you where to place the hot bags, and beseech you to recur to the rubbing.

Some observations upon the employment of stimuli may not be out of place here. The practice which has now been recommended, is essentially *non-stimulant*, and yet it will, under certain circumstances, admit of the employment of stimuli. These are in cases of drunkards, and of relapses, after a moderate re-action. In the first, during the employment of the cupping, the calomel, &c. if the patient do not very soon show some amendment, it is advisable to resort to something that will aid a debilitated stomach in coming up to a proper standard of sensibility. Hence, a strong infusion of Cayenne pepper and cloves, a table-spoonful of which to the pint of boiling water, to which, in some cases may be added a little of the spt. camphoræ, act as a cordial stimulant, highly beneficial. Of this tea he may take one or two table-spoonfuls every ten minutes, being allowed afterwards to rinse his mouth with cold water, but not to swallow it. The quantity allowed must be regulated by the effect upon the pulse. In addition to this, or sometimes as a substitute, hot brandy-toddy may be given. The carbonate of ammonia is apt to excite vomiting.

In the second case, viz. when the patient has been aroused from the torpid state of collapse, and has had bilious evacuations, showing a return of arterial action, if arising from previous debility, neglect, or feebleness of constitution, he again begin to sink, the same or milder stimuli, in smaller doses, however, may be used to advantage.

In some cases of this nature, the essence of beef, seasoned with salt and Cayenne pepper, is very grateful and restores the strength. In regard to the diet of a patient recovering from an attack of this strange affection, it is difficult to name any article that the patient will express a desire for; and in the more malignant cases, he will lie, for several days, in a state almost resembling that of the hibernating animals—he wants nothing—complains of nothing, and feels nothing. If watched carefully during this time, and the indications are met as they arise, there is no danger to be apprehended; nature will at length speak for herself; and as far as I have observed, it is generally safe to gratify the particular whim of the patient in any little matter he may desire. Hot green tea is highly grateful; cold lemonade, barley-water, chicken-water, oat-meal gruel, sago, arrow-root, mush and milk, &c., may afford us a choice, that will meet the wants of almost every case. But one thing ought to be remembered, as a general rule, that until the restoration of the *biliary and urinary secretions*, no nourishment can be wanted, and none should be allowed. The truth of this is evident in the fact that it is seldom or never called for by the patient, and his friends had better not anticipate the call. Before concluding these remarks, I will insert from the day-book the comparative results of the practice pursued, under the direction of the physician-in-chief, in the hospital, prior to the adoption of my practice, and of that which I introduced, upon the principles before explained. From July 27th to August 6th, fourteen cholera patients were received into the hospital; of these, five were cured and nine died. From August 7th inclusively, to August 21st, twenty-eight cases occurred, of these twenty were cured and eight died. Both computations have been made, by excluding all cases not choleric, and in each, some of the cases were moribund when received.

14. *Two Cases of Accidents from the Admission of Air into the Veins during Surgical Operations.* By John C. Warren, M. D., Professor of Anatomy and Surgery in Harvard University.—Some professional men have expressed doubts as to the accidental admission of air into the veins during surgical operations. Such doubts appeared well founded when the occurrence first attracted the attention of surgeons; especially on considering that veins about the neck were so very often wounded in the removal of tumors; and that some of them, as the external jugular, are frequently opened for the purpose of taking blood, without any unfavorable consequences.

Not long since I had evidence of such cases in two of my own patients within no great distance of time from each other. The certainty of such accidents, and the possibility of their frequent occurrence, have led me to consider it a matter of duty to state them publicly, for the satisfaction and government of other surgeons. It seems to me remarkable, that nothing of the kind before occurred in my own practice, nor in that of my father and predecessor in a long and active surgical career.

Case I. Mr. William Burrill, of Salem, aged 60, was admitted into the Massachusetts General Hospital, on the 16th Oct. 1830. He had a cancerous affection of the left side of the face and neck, of the extent of three or four inches diameter. It was hard at the edges, of a livid red color, ulcerated in the centre, very offensive, very painful, and had made an impression on the general health. The parotid gland, the submaxillary, the sublingual, and all the textures, excepting the bone, were involved in the complaint. The lower jaw itself was thought to be diseased at first, but it afterwards appeared that it was not so. In so bad a state of things, I felt very little hope of being able to eradicate the disease, and would not have attempted any operation had not the patient solicited it.

Considering the extent of the disease, that important blood-vessels would be divided, namely, the facial and sublingual arteries, probably the temporal, and even the external carotid, I thought it best to begin by securing the carotid trunk. An incision for this purpose was begun opposite the thyroid cartilage, and carried two inches downwards. The platysma muscle was divided; the edge of the mastoid exposed and dissected. Thus far, only a few drops of blood were discharged. The face of the sheath of the great vessels was a little uncovered, when a small effusion of venous blood appeared under the knife, and checked the operation. At that instant a very distinct sound was heard, like the passage of air through water. A few bubbles were seen in the venous blood, the flow of which was immediately arrested by applying a finger on the part. The patient exclaimed "I am faint." On regarding his countenance, it was not pale, but livid, almost black, and the muscles agitated by a convulsive motion. The respiration became deep, labored, and stertorous, like that of apoplexy. Committing the compression of the vein to Dr. Hayward, who assisted me, I examined the pulse at the wrist, found it distinct, but very slow. The wound not bleeding, and very little blood having been lost, I directly opened the temporal artery, and the blood poured from it with great freedom. As it flowed, the respiration became more frequent and less laborious; the pulse at the wrist more natural. The leaden color in the cheeks assumed a reddish tinge; and the alarming character of the symptoms was evidently diminished. About twenty minutes elapsed during these changes. At the end of half an hour I judged it safe to remove the patient to his bed, where he lay in a state of insensibility for two hours; at the expiration of which he awakened as from sleep, still breathing like an appoplectic. The night was passed without any accident, and on the following morning he was as well as usual, with the exception of a moderate soreness over the thorax, and a headache.

In seven days after the accident described above, the operation was performed without tying the carotid artery.

The diseased parts were included in an elliptical incision, extended from the lobe of the ear to the upper part of the neck, and including the submaxillary, the sublingual and parotid glands, all of them in a morbid and disorganized state. The os maxillare inferius was not diseased. The hæmorrhage was copious; but readily arrested, with the exception of that from a large vein, which from its depth under the jaw, could not be distinguished so as to admit the application of a ligature, and was therefore compressed by a sponge. The veins below the wound were compressed by Dr. Hayward during this operation. The patient experienced a slight faintness, which soon passed off. He had no bad symptoms, and on the 10th of December, the wound being nearly healed, he requested his discharge, which was granted.

Case II.—Nancy Bunker, of Trenton, in Maine, married, her age 33. Three years since, she noticed a hardness in the right breast, which increased till it involved the whole gland in a tumor, very hard, moveable, yet obviously connected with the pectoral muscle by a morbid adhesion. The nipple is drawn in. The axilla is occupied by a considerable tumor of a globular form, and quite hard. The disease has been accompanied during the last year with very constant lancinating pains. The patient is desirous of an operation; has a strong conviction that she shall not recover; yet is perfectly tranquil and resigned.

On a careful examination of the tumor, it seemed that the whole of the diseased parts could be removed, and it being thought that the patient would thus have a chance for life, and that if the disease re-appeared, her sufferings would be less than if the gland were allowed to remain. The operation was performed on the 24th December, 1831.

The patient sat in a chair. The right arm was extended, raised above a horizontal line, in order to give tension to the skin, and permit access to the arm-pit—and was supported in this position by an assistant. The skin on the surface of the breast, with the diseased nipple, were included in an oval incision, the breast was dissected from the pectoral muscle, and left connected with the axillary glands, while the extirpation of these glands was effected. As they adhered to the axillary vessels they were cautiously detached by dissection, and by insinuating the finger where the cellular substance was loose, between the tumor and the great vein. This separation was nearly effected—only a slight connexion still existing at either extremity of the tumor. Proceeding to separate it, at the outer part of the axilla, a vein was divided and a small quantity of venous blood discharged. This obscured the parts at that point, and the knife was therefore carried to the other extremity of the tumor. Scarcely was this done when the patient struggled, and on regarding her, I perceived her complexion to be a livid pale color, and at the same instant the bubbling or clucking noise was heard, though indistinctly, but the place from which it issued was not visible, the surrounding skin and fat having fallen over it, at the moment of the transfer of the knife. Directly, the axilla was compressed—the patient became insensible, breathing in a distressed manner, as in apoplexy. The tumor was at once separated. The posture of the patient was changed, and she was supported by those around. Some brandy was poured down, and ammonia was introduced into the nostrils. The pulse, however, became less distinct every instant. Cloths dipped in hot water were thrown over the extremities. Strong frictions were applied to the chest and to all parts of the body. Considerable quantities of brandy were again poured down the throat. At this moment the livid color of the cheeks gave place to a suffusion of vermilion red, and no glow in the cheek of a youthful beauty ever gave one so much pleasure as that flush. I was turning to the class who watched the different operations with intense anxiety, to say, the "danger is over," but checked myself, and continued the efforts. But the flush soon passed off; the lividity re-appeared; the respiration became more feeble; pulse at the wrist scarcely perceptible; and notwithstanding the redoubled applications of external heat and moisture, the extremities and the whole body cooled rapidly, and presently the respiration ceased.

As a last effort, I opened the larynx and put in operation the inflation of the lungs by a bellows, in a very speedy and perfect manner—imitating the movements of inspiration and expiration with great exactness—continuing the general application of heat and frictions to the whole surface. These administrations were continued for about twenty minutes longer, without any encouraging appearances. At the end of this time, I perceived there was no remaining hope of the restoration of my patient to life. The friends heing anxious to take advantage of a vessel then sailing for their home, the body was soon after removed, and no opportunity afforded for examination.

The effect of the entrance of air into the blood-vessels appear to have been known to Lieutaud Morgagni, and other distinguished pathologists, but the danger of such an occurrence in surgical operations does not seem to have been adverted to, until the operation of M. Dupuytren, in which the admission of air through the external jugular proved suddenly fatal. Since the publication of this fact, the occurrence has presented itself to many surgeons in great Britain and this country.

A natural scepticism in regard to these accidents has arisen from not considering the peculiar action of the auricles of the heart. How, it is asked, can air force itself into the veins, which are already filled with blood, and at the moment this fluid is discharging itself from an aperture in the vessel? The possibility of the accident will be however admitted, on recollecting that the auricles act not only like an expelling syringe, when they drive the blood into the ventricles, but that they have the power of suction, when they dilate themselves, thus sucking the blood from the two cavæ. This suction power of the auricles explains what would otherwise be unintelligible. The movement of blood through the large inactive veins near the heart.

There remains another difficulty. Why do not the sides of these veins collapse when the blood is pumped from them by the auricle? and if they do thus collapse, how can air be drawn in through a small aperture in one of these vessels? This objection has been removed by M. Berard, who has shown that the large veins near the heart are protected by fasciæ, connected to the coats of the veins by cellular substance. The fasciæ themselves are attached to bones, in such way as to prevent their collapsing on the veins. Further, it may sometimes happen that the coats of a vein assume a morbid structure, which gives them an unhealthy rigidity, and in this manner prevents their collapse. This occurred to M. Dupuytren, as I am informed by my friend Dr. Lodge, who was in Paris at the time. M. Dupuytren, being about to divide a large varicose saphena vein, expressed some apprehension that air might be admitted, and that the result would be fatal. The vein was divided, the peculiar sound of the entrance of air was heard, and the patient expired.

In the first of the cases above related, the vein opened was a small vein crossing the neck from the median external jugular to the great internal jugular. At least I presume this to have been the vessel; though there can be no certainty of its identity, the incision in the neck being small; the parts being not much uncovered, and the sheath of the internal jugular not opened. This small vein, stretched across the neck, was kept tense by its attachment to fixed veins at each extremity, and would thus be in a favorable position for the admission of air on the suction of the auricle.

The vein opened in the second case was the subscapular. It did not seem to be large, though perfectly visible before it was cut—and the point of incision was at a sensible distance from the great axillary vein—say nearly an inch. The dissection had separated it from the surrounding parts in a considerable degree. The axillary cavity was extensively dissected; so that the attachments of the fasciæ covering the great vein must have been much relaxed.

Here then was a small vein at some distance from the heart, dissected from the surrounding parts; and its receiving vein also dissected. The coats of the vein were not visibly diseased. The explanations of M. Berard will not therefore apply. The cause of the phenomenon in this case is to be sought in the position of the arm. The limb was extended and elevated; in consequence of which the axillary vein was in a state of extreme tension. The subscapular vein was also kept tense by the chain of axillary glands, and by the weight of the depending breast; for this organ had not been separated from the glands, in order that they might be drawn down by it and exposed.

The possibility of these accidents, under circumstances like those above, where there could be so little reason to fear them, must be a cause of anxiety to operators, in the removal of tumors from the neck and arm-pit; and I know of no effectual means to guard against them. *Pressure* on the vessels intervening between the disease and the heart, would often be impracticable; and where it could be applied, the tension of the fasciæ would generally render it abortive. Causing the patient to *expire* the air from the lungs could only be practised for a moment. *Change of position*, by relaxing the vessels, would do something; yet the state of tension must in many cases be resumed, in order to carry on the operation. The immediate compression of the vessel on the appearance of the accident, might sometimes save the patient from death, though not from very threatening appearances. For in the first place the patient's life was preserved: but although the accident was instantaneously arrested, he was saved with difficulty.

On a view of all these considerations, it appears prudent to suspend an operation in the vicinity of the heart, at the instant of appearance of venous blood from a suspicious point; and to compress the vessel, that time may be had for observing whether dangerous symptoms are likely to arise, and if these actually appear, we should directly resort to the means spoken of. First, compress the orifice of the bleeding vein with the utmost care. Second, apply pressure on the veins between the wound and the heart. Third, relax the part in which the vein is seated. Fourth, the patient may be directed to expire the air from the lungs.

The means to be pursued for saving life, after air has been admitted, have been stated in the history of these two cases, and I know of none more effectual than were adopted. The opening of the temporal artery gave great relief to case I. It was not resorted to in case II., because the patient had already lost as much blood as she could spare during the operation.

16. *A Letter on Cholera, from* Professor Chapman, *to Dr. Tyler*, —My Dear Sir—I have delayed to answer your letter till I had formed some decision as to the nature and treatment of the Pestilential Cholera which is now prevailing. These are points on which so much difference of opinion existed, that I found it impossible to make up my mind as to them, without the light of actual observation and experience. I have now seen the disease sufficiently to enable me to arrive at satisfactory, and I trust, just conclusions on the subject. But I can present in the narrow compass of a letter, only a very concise and imperfect exhibition of my views; and indeed, such are my incessant occupations, that I have scarcely leisure to execute even this slight sketch.

The disease, *wholly independent of contagion*, is caused by an epidemic agency, of which we know nothing with certainty. It is not improbable, however, that it is owing to an aeriform poison, which acting through the medium of the stomach on the ganglionic nerves, so impairs that systemt that its functions are in a greater or less degree suspended. As always happens, where sensorial or nervous influence is withheld, there is, in this case, a recession of blood from the periphery of the body, and correspondent accumulations of it in the deep seated vessels, subversive of the proper distribution of it in the circulation, attended by a vitiation or suppression of the secretions. This, in a word, is my theory of the disease, the truth of which, I think, is sustained by the symptoms, the phenomena on dissection, and the mode of cure.

It is generally held here, that cholera is almost uniformly preceded by considerable disturbances of the alimentary canal, by nausea or purging, or the two united.—That affections of this sort are very common in the city and elsewhere, cannot be denied. But whether they constitute the preliminary stage of the disease is very doubtful. It seems to me they onght rather to be considered as a condition, arising from distinct source of irritation predisposing to the disease. Can it be credited, that a cause ultimately operating so powerfully as that of cholera, should endure for three or four or five days, merely teasing in this slight manner, the stomach or bowels? The transition from these mild and lingering affections, to the explosion of cholera in its fullest force, is far too sudden and violent, to suppose that they are one and the same disease varied only by stages. I know not the analogies by which the hypothesis can be supported. Nor is this preclusive indisposition mentioned by any of the writers on Asiatic cholera whom I have consulted. It is scarcely to be presumed, that so prominent a fact, had it an existence, could have possibly escaped the attention of these very able and experienced historians of the disease.

Being attached to armies, and more particularly from their position in Hospitals, they enjoyed the best and peculiar advantages, for accurate and discriminating observations. It was first noticed, and promulgated by some of the British publications, though not sanctioned by all, and from a similar coincidence of such derangements with the epidemic in this country, the notion has been espoused by us. Be it as it may, these disorders should at once be removed, as they are apt, at all events, to invite an attack of cholera. They do not differ from the ordinary complaints of the season, and require no peculiar management.

Genuine cholera, for the most part, comes on with little or no premonition. The earliest symptoms are complaints of load and oppression, and anxiety about the præcordia, with an internal sense of heat, referrible to the stomach or bowels, with great thirst and a whitish tongue, and at the same time, the head is confused, the expression of countenance haggard, accompanied by slight nervous tremors, muscular weakness, cool skin, and either a quick and somewhat feeble, or a full and struggling pulse. Copious evacuations upwards and downwards, of a fluid resembling dirty or turbid rice-water, with floculi mixed in it, soon occur, followed by cramps or spasms of the muscles of the extremities and abdomen. These are seldom so violent as has been represented, and never extend to the alimentary canal. An aggravation of the preceding symptoms rapidly takes place, and in an hour or more, the tongue becomes icy cold, the skin more chilled and sodden, though feeling hot to the patient, coveree with dewy viscid perspiration, the hands shrivelled or wilted, as if macerated, the nails of the fingers blue, the pulse scarcely or not at all perceptible, the face sunken, especially the eyes, around which is a dark circle. The color gradually diffuses itself more or less over the entire surface, partaking of the varied shades of lividness, from a saturine to a bluish or blackish hue. During this period the thirst is intense, the heat of the stomach in some instances is increased to a burning sensation, the respiration greatly embarrassed, the air expired cold, the voice low, or whispering and plaintive, the diaphragm convulsed, and there is a total suppression of the urinary and other secretions. Discharges from the alimentary canal, and the spasms, likewise cease or are much diminished. The intellectual faculties, though obtuse, are seldom otherwise affected, and in some instances, their integrity is throughout preserved. Death ultimately takes place in a sort of tranquil stupor, or with indescribable jactitation and distress, the latter state being by far the more common.

As I have briefly described the disease, such is the tenor of its character and progress, though occasionally diversified in some respects. Thus I have seen its accession as sudden as the electric shock, and have met with cases without spasms, or vomiting or purging. Many other anomalies might be mentioned, could I indulge in such details. The disease may be properly divided, in most instances, into two stages—that of aggression and collapse.

Called at the commencement of an attack, unless there is an extreme depression, I bleed freely from the arm, and uniformly cup the epigastrium, and give calomel largely, combined or not with opium, according to the severity of the spasms. The case will almost invariably yield to these remedies, and we have no further trouble concerning it. But where the attack is confirmed, or in other words the state of collapse exists, the difficulties of management are vastly increased, and the practice is somewhat different. The first step under such circumstances is to puke actively with tepid salt and water, a tumblerful at a time. This usually settles the stomach, allays thirst, produces some degree of re-action, a stronger pulse, increased warmth of surface, and a resolution of the spasms. Co-operating in the same design of arousing the vital forces, and exciting the skin particularly, the body and extremities may be rubbed with warm flannels. Let a vein be then opened, and if the blood flows freely, take a large quantity, and especially should the pulse rise and the blood become florid. But where the reverse happens, or you have slowly to coax out the blood, or the pulse is sensibly weakened by the loss of it, stop the operation, and apply twenty or thirty cups to the abdomen, including the epigastrium, which, though they may not draw much blood, are eminently serviceable as revellents—the cups are to be succeeded by a blister to the same parts. Calomel is next to be given in the dose of five, ten or twenty grains, frequently repeated, till the aggregate amounts to about a drachm, and then worked off with a table spoonful of castor oil. As the result of these means there are commonly bilious evacuations, discharges of urine, and other proofs of the restoration of secretory power. Little more is demanded than what has been mentioned. I have however, sometimes known, though rarely, at this point of the case, irritability of the stomach to return, with the appearance of approaching exhaustion, in which event stimuli are to be resorted to—the best of which are, a strong infusion of Cayenne pepper, or clove tea, or spirits of camphor, or the aromatic spirits of amonia, or mint julep. Bnt they are cautiously to be administered, and in small potions, of they are instantly rejected, or they overwhelm the energies of life, or more slowly induce typhoid prostration.

Drink is sometimes vehemently solicited, particularly in the height of the attack, and the instinctive desire for cold water, or even for ice, may be gratified in moderation. The proper nourishment in convalescence, the only time when any is wanted or to be allowed, is chicken water or beef tea, rendered agreeably pungent with Cayenne pepper. Thus I have hastily laid before you an outline of my mode of managing this disease. It may be observed that, with scarcely an exception, it is depletory or evacuant. Deluded by false appearances of debility in the disease, and still more by the weight of authority, I adopted, when it first broke out among us, in common with my medical friends, a course of practice in conformity with such an impression—and most disastrous was the issue. Nearly every patient, amounting to five or six, in different hospitals, died. The prominent indications seemed to call for heat to the surface, and the internal exhibition of the diffusible excitants. Every variety of bath, warm water, vapor, heated air and topical applications of hot sand or oats, or salt, &c. were used, and also frictions with the spirits of turpentine alone, or united with camphorated mercurial ointment, and other articles. Brandy, ether, camphor, vol. alkali, &c. &c., were in succession tried, and the whole of these means with no other effect than an inconsiderable exasperation. The suffering, indeed, induced, was as great as I have ever witnessed from the application of any remedial process. No practical lesson is more important than that in the cure of this disease, all such appliances and medicines are mischievous, till evacuations are premised, and then to be most discreetly directed. The system previously, is utterly intolerant of them, and I have found it better to expose the patient naked to cool air, than to cover him over even with a blanket.

It were easy to acquaint you with divers other methods of treating this epidemic, or to enumerate a number of special remedies that have been proposed. Dismayed, as it were, from the fearful character of the disease, practitioners have been too prone in its treatment, to abandon their principles and well tried remedies, in analogous cases, to seek a resource in specifics and nostrums.

I do not mean to vaunt of my success, but on a fair comparison of all that I have seen attempted, I am led to an unqualified preference of this plan. It cannot be charged with being tentative or empirical—is deduced, on the contrary, from established views of pathology and therapeutics, and is sanctioned in most of its features by the lengthened and concurrent experienced and authoritative writers on the disease in India. Many may be cured by it, and some will sink under the force of the attack in despite of your efforts. The case not being too far advanced, a triumph over the disease is pretty certain. Cholera is, on the whole, more tractable than yellow fever, or the winter pestilence which devastated our country during the late war.

Ever, my dear sir, yours, most truly, NATHAN CHAPMAN

*Philadelphia, August* 13, 1832.

17. *Dr. Clarke's Algid Fever of Smyrna compared with Dr. Barry's Blue Cholera.*—Sir,—In the October Number (1826) of the Medico-Chirurgical Review, there is published a paper written by Dr. Clarke, descriptive of a strange disease that was epidemic at Smyrna, in 1825–6, and which bears so striking a likeness to the worst cases of blue cholera, that the following passage may be worth transcribing at the present moment.

"There is no shivering, nor is the slightest sensation of cold complained of. The pulse, if felt at all, is thready and rapid, disappearing under the slightest pressure of the finger. There is, more usually, a total want of pulsation in all tangible vessels, and even in the region of the heart. The countenance, in some cases, is livid, and betrays unspeakable anguish. The features are withered and shrunk. The intellectual powers are, for the most part, but little impaired, and there is frequently a command of muscular exertion a calm composure and self-possession, which would lead any one, unacquainted with the patient's situation to believe him free from complaint. But when the fingers are applied to the wrist, and the pulse is sought in vain—when the more than deadly coldness of the surface is perceived, the illusion vanishes. In this state of equivocal existence, he continues from ten to eighteen or twenty-four hours, and, in some cases, much longer, when, if he survives the paroxysm by the persevering application of remedies, and the re-active energies of the system, an increase of temperature takes place, which gradually acquires different degrees of intensity, and occasionally requires to be moderated."—MEDICUS.

The above description is so perfectly identical with some of the very *worst forms* of the present epidemic, that we think it is well worthy of a place here.—*Medico-Chirurg. Rev.*

18. *Two Cases of Dysentery, accompanied by an unusual Affection of the Skin.* Communicated in a letter to the Editors by A. B. Price, M. D.

GENTLEMEN: I am induced to send you this communication, because I have not seen a similar case recorded, and from a full belief, that it is the duty of every member of the profession to contribute his mite to the general stock of knowledge.

The following history of two anomalous cases of Dysentery, occurred in this vicinity, and was related to me by the father of the subjects of the case; and may be relied upon as strictly correct, as he is a man of unblemished character.

In the summer of 1824, the flux prevailed in this neighborhood generally. Among the first cases, were two of his children, who were attacked severely, though one more severely than the other. The treatment was domestic; consisting of a cathartic, at the outset, followed by a liberal use of a decoction of the dewberry in milk, and slippery elm emulsion. In a few days after this plan was adopted, the discharge from the intestines suddenly ceased, and the skin of the body and extremities, in patches of some size, assumed the appearance of a blistered surface; from which proceeded a thin discharge, of a pale, milky appearance, which ran down the legs in great profusion. The little patients were in an uncomfortable situation; they could neither stand, lie, nor sit long at a time; the discharge from the skin bore an exact relation to the severity of the discharge from the bowels, in both cases; it gradually diminished, and in a few days ceased. The children convalesced from the time that it commenced running. Was not this a metastasis of Dysentery?—if not, what was it?

The above is the substance of what was related to me by the father of the children.

---

19. *Elements of Chemical Philosophy, on the basis of Reid, comprizing the Rudiments of that Science, &c.* By Thomas D. Mitchell, M. D. Corey and Fairbank—pp. 600.—We have just received from the publishers a copy of the above work, and from a hasty view of it are disposed to entertain a very favorable opinion of it as a text book for students of Medicine, and a book of reference for general readers. We shall notice it more particularly in our next number. It is published in a very superior style of execution highly creditable to the enterprizing publishers.

